# Operationalizing the physiological state (P) in nonlinear behavioral systems: a measurement framework for state-dependent dynamics

**DOI:** 10.3389/fpsyg.2026.1873356

**Published:** 2026-10-14

**Authors:** Yoandra M. Gomez Uncu

**Affiliations:** Beautiful Minds ABA Services, Fort Myers, FL, United States

**Keywords:** latent-state estimation, nonlinear state-space modeling, nonlinear systems, physiological accessibility, physiological state, psychophysiological measurement, state-dependent behavior

## Abstract

Behavioral outcomes may vary within the same individual even when measured environmental conditions appear comparable. To test whether an organism’s changing physiological condition provides information about such variation, this article proposes an operational measurement framework for Physiological State (*P). P* is defined as a latent, multidimensional, and time-dependent configuration generated by ongoing Physiological Organization. Antecedent conditions (*A*) and consequence-related conditions (*C*) remain external environmental variables, whereas behavior (*B*) is modeled as a separate downstream outcome. Rather than uniquely determining behavior, *P* influences Physiological Accessibility—the state-dependent availability of existing Adaptive Capacities for recruitment and use—and thereby constrains behavioral expression. The construct is distinguished from emotional and cognitive constructs, subjective reports, observable behavior, stress, allostatic load, and individual physiological indicators. Operationalizing requires prespecifying the target behavior and timescale; selecting reliable, sensitive, feasible, and nonredundant physiological indicators; aligning and preprocessing observations; deriving dynamic features; integrating those features through an identifiable latent-state model; representing estimated uncertainty; and conducting prospective validation. The Thirteen Physiological Systems and Seven Layers of Physiological State guide physiological coverage and interpretation but do not correspond one-to-one with the latent dimensions. In a candidate nonlinear state-space implementation, physiological observations, *Y*(t), provide evidence for estimating *P*(t), while state evolution depends on preceding *P,* current *A*, and relevant prior *C*. Filtered estimates use only information available through the prediction time. Because *B* remains outside the physiological observation vector and is evaluated prospectively as *B*(t + Δ), the framework avoids circularly defining the state by the outcome it is intended to predict. Empirical support would require reliable, interpretable, and temporally coherent state estimates that demonstrate reproducible prospective and incremental value beyond environmental variables, prior behavior, isolated physiological indicators, established single constructs, simple physiological composites, and appropriate linear or lower-dimensional alternatives. Failure to obtain identifiable state estimates or incremental prospective information about *B* would count against the proposed operationalization. The framework is theoretically and methodologically developmental and should not be interpreted as a validated instrument, diagnostic measure, or universal physiological score.

## 1 Introduction

### 1.1 Limitations of simplified linear representations of behavior

Behavioral science examines relations between environmental conditions and behavioral outcomes. Contingency-based approaches identify functional relations among antecedent conditions, behavioral responses, and consequences (Skinner, 1953; Cooper et al., 2020). Although these approaches are not inherently linear or deterministic, simplified input–output representations may imply stable or proportional environmental behavioral relations, inadequately capturing prior-state dependence, time-varying interactions, thresholds, and emergent dynamics (Thelen and Smith, 1994; Kelso, 1995; Sterling, 2012).

Intraindividual variability further challenges stable input–output representations. Behavioral and psychological measures may change within the same individual over time, and between-person associations need not reproduce within-person dynamics (Ram and Gerstorf, 2009; Fisher et al., 2018). Comparable measured environmental conditions may therefore yield different outcomes. Such differences do not establish the existence or causal influence of *P*; they may reflect measurement error, unmeasured conditions, prior behavior, learning history, sampling limitations, or other unmodeled influences.

The relevant question is whether an independently estimated Physiological State accounts for this variation reproducibly and distinctly. The framework asks whether *P* provides prospective, incremental information about subsequent behavior beyond measured environmental conditions, prior behavior, and simpler physiological models while accounting for measurement uncertainty. The article develops an architecture for estimating *P* from physiological evidence and testing its contribution within a nonlinear behavioral system.

### 1.2 Physiology as an underoperationalized variable

Psychophysiology and integrative physiology provide established methods for examining physiological processes and behavior (Cacioppo et al., 2017; Hall, 2016). However, physiological information is commonly represented by individual indicators or narrower constructs such as arousal, stress, and allostatic load rather than a unified organism-level state within a behavior system. Models describe adaptive, history-dependent regulation (Sterling, 2012), but do not operationalize *P* or this architecture.

The framework distinguishes Physiological Organization from Physiological State. Physiological Organization is the continuous interaction and reorganization of physiological systems and regulatory domains. Physiological State, *P*(t), is the latent, multidimensional, time-dependent configuration generated at a given moment (Gomez Uncu, 2025b, 2026a). Thus, organization remains dynamic while the state is estimated at specified observation times and compared across persons, contexts, or periods, without treating any single measurement as a complete representation of that state by itself.

Construct boundaries are essential. *P* is not equivalent to stress, arousal, allostatic load, emotion, cognition, subjective experience, behavior, or any single physiological indicator. Physiological observations and dynamic features provide partial, fallible evidence for estimating *P*. Processes associated with emotion or cognition may contribute evidence, but subjective reports, cognitive-task performance, and B remain outside the physiological observation vector. Antecedent- and consequence-related conditions, A and C, also remain external to P.

### 1.3 Need for nonlinear, state-dependent systems

Dynamical systems approaches represent behavior as the product of interacting components with nonlinear, time-dependent relations (Kelso, 1995). These principles make a state-dependent physiological framework plausible but do not validate its constructs or architecture.

Behavioral variability may reflect motivating operations, stimulus control, environmental contingencies, learning history, or measurement error (Michael, 1993; Laraway et al., 2003; Cooper et al., 2020). The contribution of *P* must therefore be tested against models incorporating these factors. Under comparable measured conditions, independently estimated *P*(t) should improve the prospective, out-of-sample prediction of *B*(*t* + Δ) beyond environmental variables, motivating operations, prior behavior, and simpler physiological representations while accounting for measurement uncertainty. Failure to show reproducible incremental information would count against the proposed operationalization of P.

The framework separates state estimation from behavior evaluation: Physiological observations estimate *P,* whereas *B* remains a downstream outcome. *P* influences Physiological Accessibility, constraining which Adaptive Capacities can be recruited and expressed behaviorally (Gomez Uncu, 2025b, 2026a). This is state dependence, not physiological determinism. Antecedent conditions (*A*) and consequence-related conditions (*C*) remain external to *P*: current A and relevant prior C may contribute to the evolution of *P*, current *P* may modulate functional effects of environmental conditions, and B may alter subsequent conditions.

Environmental, contextual, social, cultural, symbolic, developmental, historical, and learning-related influences remain distinct from *P*. Within the framework, their behavioral effects are mediated through *P*, although changes in *P* do not necessarily produce changes in observable behavior.

### 1.4 Objective, contribution, and scope

This article translates earlier theory into an operational, computational architecture for estimating and evaluating *P* within a nonlinear organism–environment system (Gomez Uncu, 2025b, 2026a, 2026b).

Its primary contribution is a measurement-development architecture, not a fixed physiological score or a predetermined set of state dimensions. It establishes *P’s* boundaries; criteria for selecting indicators; procedure for temporal alignment, preprocessing, and dynamic feature derivation; methods for multidimensional latent-state estimation and uncertainty representation; a candidate nonlinear state-space implementation; and procedures for model comparison and prospective validation. The number and meaning of latent dimensions must be theoretically justified and empirically supported for the population, behavior, and timescale investigated.

The architecture separates *P* from its generating Physiological Organization, the evidence used to estimate it, external variables *A* and *C*, and outcome *B*, preventing retrospective definition by the behavior predicted. It should therefore be evaluated as a proposed architecture, not as a validated instrument, universal score, diagnostic tool, or intervention protocol. Its value depends on identifiable, reliable, and interpretable state estimates demonstrating prospective validity against prespecified simpler alternatives.

## 2 System definition: nonlinear physiology–behavior model

This section formalizes the Nonlinear Physiology–Behavior Equation and specifies the role of Physiological State (*P*) within the system. Earlier formulations introduce the framework’s dynamic and regulatory principles (Gomez Uncu, 2025b, 2026a); the present article develops the measurement specifications required to estimate *P* and evaluate its proposed relation to behavior. Established physiology describes multisystem coordination (Hall, 2016), allostatic theory describes adaptive and predictive regulation (Sterling, 2012), and dynamical-systems theory describes nonlinear, time-dependent interaction (Kelso, 1995). These bodies of work provide the broader scientific basis for the framework but do not independently validate its variables, proposed dependencies, or measurement architecture.

At the conceptual level, the principal dependencies are expressed as follows:


dP(t)/dt=F(A(t),P(t),C(t)) (1)

B(t)=f(P(t)) (2)

Equation 1 represents the rate of change of *P*(t) as a function of time-indexed antecedent conditions, *A*(t); the current physiological state *P*(t); and consequence-related conditions *C*(t). Because *P*(t) is multidimensional, *F* is vector-valued: its output must have the same dimensionality as *P*(t) and must represent change in physiological state per unit of time. Equation 2 represents the proposed pathway from *P*(t) to its observable downstream expression, *B*(t). It is a reduced conceptual relation rather than a complete behavioral model. Equations 1, 2 specify the proposed dependency structure but do not provide the observation and state-transition model needed to estimate latent *P*. The four primary system variables are therefore *A, P, C*, and *B*. Physiological Organization is not an additional state variable; it is the continuous internal process that generates *P*.

### 2.1 System variables (*A, P, C,* and *B*)

The system comprises four time-indexed variables with distinct environmental, physiological, behavioral, and analytical roles:

- A(t)—Antecedent Conditions. *A*(t) comprises external environmental conditions measured within a prespecified interval preceding or contemporaneous with the estimation of *P*(t) and, in prospective tests, preceding the target behavior *B*(t + Δ). These conditions may be contextual, social, cultural, situational, task-related, or stimulus-related. *A* remains external to *P*. Although *A*(t) may contribute to change in *P*(t), the current state may modulate the individual’s sensitivity or functional response to the antecedent condition. Thus, the A ↔ P relation does not imply that P alters the physical occurrence of the antecedent event.
- P(t)—Physiological State. *P*(t) is the latent, multidimensional, and time-dependent physiological configuration present at time *t* and generated by ongoing Physiological Organization. Physiological Organization refers to the continuous internal process through which physiological systems and regulatory layers interact, coordinate, and reorganize over time. *P* is distinct from that generative process and from any individual physiological process, construct, signal, or indicator (Gomez Uncu, 2025a, 2026a). Integrative physiology and research on interoceptive signaling support the plausibility of coordinated physiological processes (Craig, 2009; Hall, 2016), but do not independently establish the framework-specific construct or architecture proposed here.
- C(t)—Consequence-Related Conditions. *C*(t) comprises external environmental events or conditions that followed an earlier behavior or organism–environment interaction and remain active or functionally relevant during the current state-estimation interval. Accordingly, *C*(t) occurs after the earlier behavior for which it functioned as a consequence but before the subsequent target behavior *B*(t + Δ). *C* remains external to *P* and is measured independently. Although *C*(t) may contribute to changes in *P*(t), the current state may modulate its functional effect. A consequence that occurs after *B*(t + Δ) belongs to a later observation interval and cannot serve as a predictor or cause of that same behavioral occurrence.
- B(t)—Behavior. *B*(t) is the observable downstream behavioral expression measured during the target observation interval. It is not uniquely determined by *P*. When behavioral expression requires an existing Adaptive Capacity, its occurrence may depend partly on whether that capacity is physiologically accessible under the current state (Gomez Uncu, 2025b, 2026a). In a prospective design, the target outcome is represented as *B*(t + Δ), where Δ is the prespecified interval between estimating *P*(t) and measuring subsequent behavior. Repeated estimation of *P* permits testing whether within-person changes in the state precede and provide prospective information about changes in behavioral expression. *B* remains analytically separate from *P* and is excluded from the physiological observations used to estimate the state.

For empirical estimation, *Y*(t) denotes the temporally aligned physiological observations and derived dynamic features used to estimate *P*(t). The resulting estimate, P̂(t), must be accompanied by an appropriate representation of uncertainty. No individual observation is equivalent to *P*. Although *A* and *C* remain outside *Y*(t), they may enter the state-transition model as external variables. *B*(t + Δ) remains a separate downstream outcome.

### 2.2 Conceptual system structure

The Non-Linear Physiology–Behavior Equation is represented conceptually as follows (Figure 1; Gomez Uncu, 2025b).

**Figure 1:**
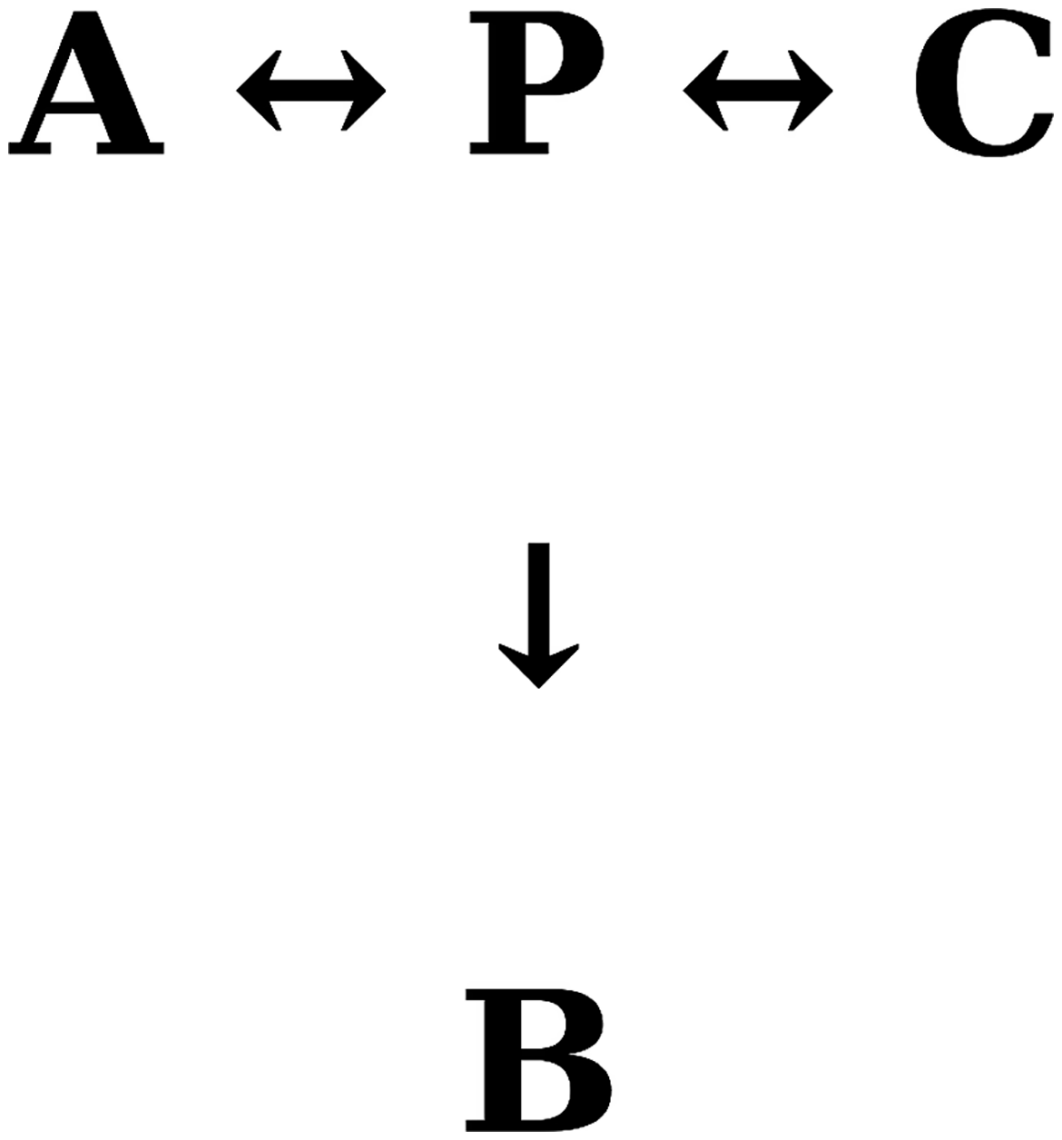
Nonlinear physiology–behavior equation (conceptual system representation). P(t) is the time-indexed latent Physiological State generated by ongoing Physiological Organization. A(t) and C(t) are external environmental conditions. The bidirectional arrows represent hypothesized coupling between A and P and between C across time; they do not establish instantaneous or reciprocal causation. Current A and relevant prior C may contribute to the subsequent evolution of P, where P may modulate their functional effects. Internal physiological processes remain within Physiological Organization and are not classified as A or C. B(t) is the observable downstream behavioral expression associated with P(t). Through Physiological Accessibility, P is hypothesized to constrain, without uniquely determining, the recruitment and expression of existing Adaptive Capacity. Environmental consequences following B may enter a later observation interval through C and may subsequently function as A, depending on their temporal and functional position. The figure is a conceptual dependency map rather than a complete causal, measurement, state-transition, or behavioral-output model.

This diagram represents proposed nonlinear interactions between *A* and *P* and between *P* and *C* within a continuously evolving regulatory system. It should be interpreted as a conceptual dependency map, not as a complete causal, statistical, or measurement model. In particular, it does not specify temporal lags, functional forms, parameters, measurement error, or person-specific variation.

The conceptual system assigns *P* two related but analytically distinct roles: it is the latent state through which environmental conditions may be physiologically integrated, and it constrains the Physiological Accessibility of capacities required for downstream behavioral expression. Regarding the first role, the framework does not assume that *A* and *C* directly or proportionally determine *B*. Instead, these external conditions interact with ongoing Physiological Organization and may contribute to changes in *P*(t) over time (Gomez Uncu, 2026a). Current *P*(t) may, in turn, modulate their functional effects. Thus, the bidirectional arrows represent hypothesized functional interactions across time rather than instantaneous interaction or established reciprocal causation.

Regarding the second role, *B* remains outside the bidirectional A ↔ P ↔ C relation and is positioned as the observable downstream expression of *P*. The resulting behavior may nevertheless alter later environmental conditions: consequences or environmental changes following B may enter subsequent interactions as *C* or *A,* depending on their temporal and functional positions. Equation 2 expresses the proposed state-to-behavior relation.The relation is state-constrained because *P* influences Physiological Accessibility, which determines the availability of existing Adaptive Capacities for recruitment and behavioral expression. It is not intended to imply that *P* uniquely determines which behavior occurs. Emotion, cognition, subjective experience, contextual conditions, and behavior remain analytically distinct from *P* and are not its latent dimensions, although their associated physiological processes may contribute evidence for estimating the state.

Because *P* changes over time, comparable measured antecedent conditions may be associated with different behavioral outcomes when they encounter different physiological states (Gomez Uncu, 2026c). Such behavioral divergence is consistent with state dependence but does not establish that different Physiological States were present. That claim requires *P* to be estimated independently from physiological evidence.

### 2.3 Functional roles of the variables

The conceptual positions of *A, P, C*, and *B* translate into three distinct analytical roles:

- Externally measured environmental inputs *A*(t) and *C*(t): Current A and relevant prior C may enter the state-transition model as measured inputs and the comparator or behavioral-outcome models as covariates. Their effects may vary over time and are not assumed to be linear or proportional. Neither variable is inferred from *P* or included among the physiological indicators used to estimate it.
- Latent central Physiological State *P*(t): Physiological Organization generates *P*(t), which is estimated from temporally aligned physiological observations and derived dynamic features. The estimate must be accompanied by an appropriate representation of uncertainty. In the transition model, preceding *P* captures prior-state dependence, whereas current *A* and relevant prior *C* may contribute to subsequent state evolution. In the behavioral-outcome model, P̂(t) may be evaluated prospectively as a predictor of *B*(t + Δ) and as a moderator of measured environmental effects. Through Physiological Accessibility, P is hypothesized to constrain which existing Adaptive Capacities are available for behavioral expression.
- Observed behavioral outcome B(t): Prospective analyses evaluate *B*(t + Δ) in relation to P̂(t), measured environmental variables, relevant history, and competing predictors. *B* must remain outside the physiological observation vector and cannot be used to select latent dimensions in a confirmatory *P–B* test; either practice would introduce circularity or data leakage. The architecture specifies no direct *B → P* pathway. Instead, environmental consequences following *B* may enter later interactions through *C* or *A*.

These analytical roles define a three-component empirical architecture:

1. Measurement: Physiological observations and derived features, represented by *Y*(t), provide the evidence from which P̂(t) is estimated.
2. State transition: Preceding *P*, current *A*, and relevant prior *C* are evaluated as contributors to the modeled evolution of the physiological state.
3. Behavioral outcome: P̂(t), measured environmental variables, relevant history, and competing predictors are evaluated prospectively in relation to *B*(t + Δ).

Separating these components permits the proposed nonlinear, state-dependent architecture to be compared with environmental-only models and simpler physiological alternatives without defining *P* from the behavior it is intended to predict.

## 3 P as a state variable

### 3.1 P as an integrated physiological state

Two distinctions define *P* as a state variable: the distinction between Physiological Organization and the state it generates, and the distinction between physiological integration and simple aggregation. Physiological Organization is the continuous process through which physiological systems and regulatory layers interact and reorganize. *P*(t), by contrast, is the latent, multidimensional, and time-dependent configuration generated by that process at time *t* (Gomez Uncu, 2025a, 2026a). Integration therefore refers to coordinated cross-system patterns and temporal dependencies, not to a simple sum of indicators, equal contributions from all physiological systems, or a universal score. Integrative physiology and interoceptive research support the plausibility of coordinated physiological processes (Craig, 2009; Hall, 2016), but do not independently establish *P* or the proposed architecture.

The construct boundaries determine what may be used to estimate *P*. The state remains distinct from *A, C, B,* emotion, cognition, subjective experience, and narrower physiological constructs such as stress, arousal, allostatic load, and interoception. Although physiological processes associated with emotional or cognitive functioning may contribute to measurement evidence, those constructs and their subjective or behavioral measures are not dimensions or indicators of P. Consequently, only physiological observations and derived physiological features may serve as indicators of the latent state.

Because P cannot be observed directly, it must be inferred from physiological evidence collected within a prespecified measurement window. Let *Y*(t) denote the vector of temporally aligned physiological observations and derived dynamic features. The general observation model is:


Y(t)=h(P(t);θ)+ε(t) (3)

The potentially nonlinear function *h* maps the latent state *P*(t) onto the physiological observations in *Y*(t); θ denotes the measurement-model parameters; and ε(t) represents measurement error and residual variation not captured by the latent state.

The resulting estimate, P̂(t), must be reported with model-appropriate uncertainty (Särkkä and Svensson, 2023). Neither its dimensionality nor the contributions of its indicators should be inferred from the number of physiological systems, regulatory layers, or available measurements; both require empirical evaluation. In prospective *P–B* tests, *B* must remain outside Y(t) and cannot contribute to the construction of P̂(t).

### 3.2 *P* as the central state variable

The centrality of *P* does not mean that it subsumes environmental, emotional, cognitive, subjective, or behavioral constructs. Rather, *P* occupies the state position connecting ongoing Physiological Organization to the physiological availability of existing capacities and, ultimately, to downstream behavioral expression. Equation 2 represents this proposed direction of dependence through a potentially nonlinear, state-constrained relation. It is not a complete statistical model of behavior.

The proposed pathway is:


$$\begin{array}{l} P(t)\to \text{Physiological Accessibility}(t)\to \text{recruitment}\;\\ \;\;\;\;\text{and}\;\mathrm{use}\;\text{of existing Adaptive Capacities}\to B(t+\Delta ). \end{array}$$

Physiological Accessibility denotes the state-dependent availability of existing Adaptive Capacities for recruitment and use. Within this framework, cognitive analysis is treated as an Adaptive Capacity rather than as a dimension or indicator of *P*; its availability for recruitment may therefore vary with Physiological Accessibility. Restricted accessibility does not indicate that a capacity has been lost, just as access to a capacity does not uniquely determine its behavioral expression. Physiological Accessibility is itself distinct from *B* and cannot be inferred solely from whether *B* occurs (Gomez Uncu, 2025a, 2026a).

For empirical evaluation, the corresponding behavioral-outcome model may be expressed as follows:


$$B(t+\Delta )∼p_{B}\;(\cdot |\;P(t),Z(t);\psi )$$ (4)

Here, p_B_ denotes the prespecified conditional distribution of the subsequent behavior outcome, B; Z(t) contains separately measured nonphysiological predictors and covariates, and ψ denotes outcome-model parameters. Depending on the research question, Z(t) may include A(t), relevant prior (C), motivating operations, learning history, prior behavior, and contextual variables. These variables remain outside (P), thereby allowing the model to test whether P(t) contributes prospective and incremental information beyond established environmental and historical predictors. If the estimated state, P̂(t), replaces latent P(t) in a two-stage analysis, the uncertainty introduced during state estimation must be represented in the behavioral-outcome analysis.

The central position assigned to (P) therefore identifies its role within the proposed pathway, not its status as the sole or sufficient cause of behavior. The model does not exclude relevant environmental, historical, or nonphysiological influences. Its empirical value depends on whether it provides reproducible information beyond these influences and beyond simpler physiological representations.

### 3.3 Temporal and dynamic properties of P

Because (P) is dynamic and time-dependent, its properties must be evaluated across repeated observations. Its evolution is not assumed to be linear or proportional to changes in environmental conditions. Instead, it may exhibit structured variability, prior-state dependence, relative stability, recovery, threshold-dependent change, or transitions among regions of the physiological state space. Dynamical-systems theory provides a basis for representing such properties mathematically but does not establish that they occur in (P) (Kelso, 1995; Strogatz, 2015).

For observations collected at discrete times (t₁, t₂, …, tₙ), a general state-transition model is as follows:


$$p(t_{k})=g(p(t_{k-1}),A(t_{k}),C(t_{k-1}),\Delta t_{k};\theta_{P})+\omega_{k}$$ (5)

In Equation 5, P(tₖ₋₁) is the preceding physiological state; A(tₖ) represents antecedent conditions measured for the transition ending at tₖ; C(tₖ₋₁) represents consequence-related conditions from the preceding interval that remain relevant to the current transition. Each implementation must prespecify whether A(tₖ) is measured at tₖ or summarized over (tₖ₋₁, tₖ]; in either case, only information available by tₖ may be used. The elapsed time between observations is Δtₖ = tₖ − tₖ₋₁, θₚ denotes the transition parameters, and ωₖ represents process variation not explained by the modeled transition.

The function (g) is the discrete-time transition map corresponding to the continuous-time function (F) in Equation 1 or a prespecified numerical approximation to that map. Equation 5 therefore translates the (A)–(P)–(C) dependencies in Equation 1 to the intervals at which observations are collected; it does not replace Equation 1. (B) remains outside the state-transition model as the downstream behavioral outcome.

Variability in physiological observations may provide structured evidence about (P), but such variability is not automatically state-related, adaptive, or dysfunctional (Sterling, 2012). Apparent changes may result from artifacts, movement, physiological rhythms, sampling or preprocessing decisions, missing data, or physiological processes unrelated to (P) (Cacioppo et al., 2017). The model therefore distinguishes observation error from state-process variation: ε(t) in Equation 3 represents measurement error or residual variation in Y(t), whereas ωₖ in Equation 5 represents unexplained variation in the evolution of the latent state.

A single P(tₖ) represents one latent state, not a trajectory. The ordered sequence {P(t₁), P(t₂), …, P(tₙ)} defines a sampled theoretical trajectory, whereas {P̂(t₁), P̂(t₂), …, P̂(tₙ)} is its estimated counterpart. Valid trajectory-level interpretation requires physiologically appropriate sampling and temporal alignment, as well as pointwise and trajectory-level representations of uncertainty.

Claims of threshold-dependent change or distinct state transitions cannot be established through visual inspection alone. They require prespecified criteria and formal comparison with linear-transition, smooth nonlinear, and nonswitching alternatives using held-out or otherwise analytically separated data.

### 3.4 Implications for measuring P

The defining properties of (P) impose six requirements on its empirical estimation:

1. Multidimensional evidence. (P) cannot be identified with an isolated physiological indicator or an equally weighted composite. Each measure provides only partial evidence and must be integrated within a multimethod design (Cacioppo et al., 2017). Simple physiological composites may nevertheless be retained as comparison models.
2. Repeated and prospective measurement. Although a single observation may inform the estimate of (P) at one moment, it cannot characterize state evolution. The sampling intervals, state-estimation windows, and the interval between P̂(t) and B(t + Δ) must therefore be prespecified. Prospective tests require filtered estimates based only on information available through time (t); smoothed estimates that incorporate later observations are retrospective and cannot support prospective prediction.
3. Formal evaluation of nonlinear dynamics. Structured variability, persistence, recovery, thresholds, and transitions should be evaluated only when they are relevant to the hypothesis and supported by the data. Claims concerning thresholds or regimes require prespecified criteria and comparison with linear-transition, smooth nonlinear, and nonswitching alternatives (Kelso, 1995; Strogatz, 2015).
4. Separation of state variation from observation error. Artifacts, movement, device drift, physiological rhythms, irregular sampling, missing data, and preprocessing decisions may produce apparent physiological changes (Cacioppo et al., 2017). Measurement protocols must therefore specify calibration procedures, artifact and missing-data procedures, temporal alignment, and the treatment of measurement error.
5. Restraint in the mechanistic interpretation. Allostatic theory describes adaptive, predictive, and anticipatory regulation (Sterling, 2012). Within the present framework, however, temporal patterns alone do not establish allostatic load redistribution or any other proposed unifying physiological mechanism (Gomez Uncu, 2025a, 2026a). Mechanistic claims require longitudinal, perturbational, or experimental evidence capable of distinguishing among alternative explanations.
6. Prespecified development and validation. Studies must define the target behavior, population, context, timescale, indicators, measurement windows, preprocessing procedures, integration method, quality controls, and person-specific baselines. Indicators should be theoretically relevant, reliable, sensitive, feasible, and nonredundant. The state estimate, P̂(t), must be accompanied by model-appropriate uncertainty (Särkkä and Svensson, 2023), and (B) must remain outside (Y(t)). Indicator selection and model development must also be separated from confirmatory behavioral validation through held-out observations, independent samples, or another prespecified procedure.

Accordingly, P̂(t) is an uncertain estimate of latent (P(t)), not an error-free observation, validated instrument, or universal physiological score (Figure 2).

**Figure 2:**
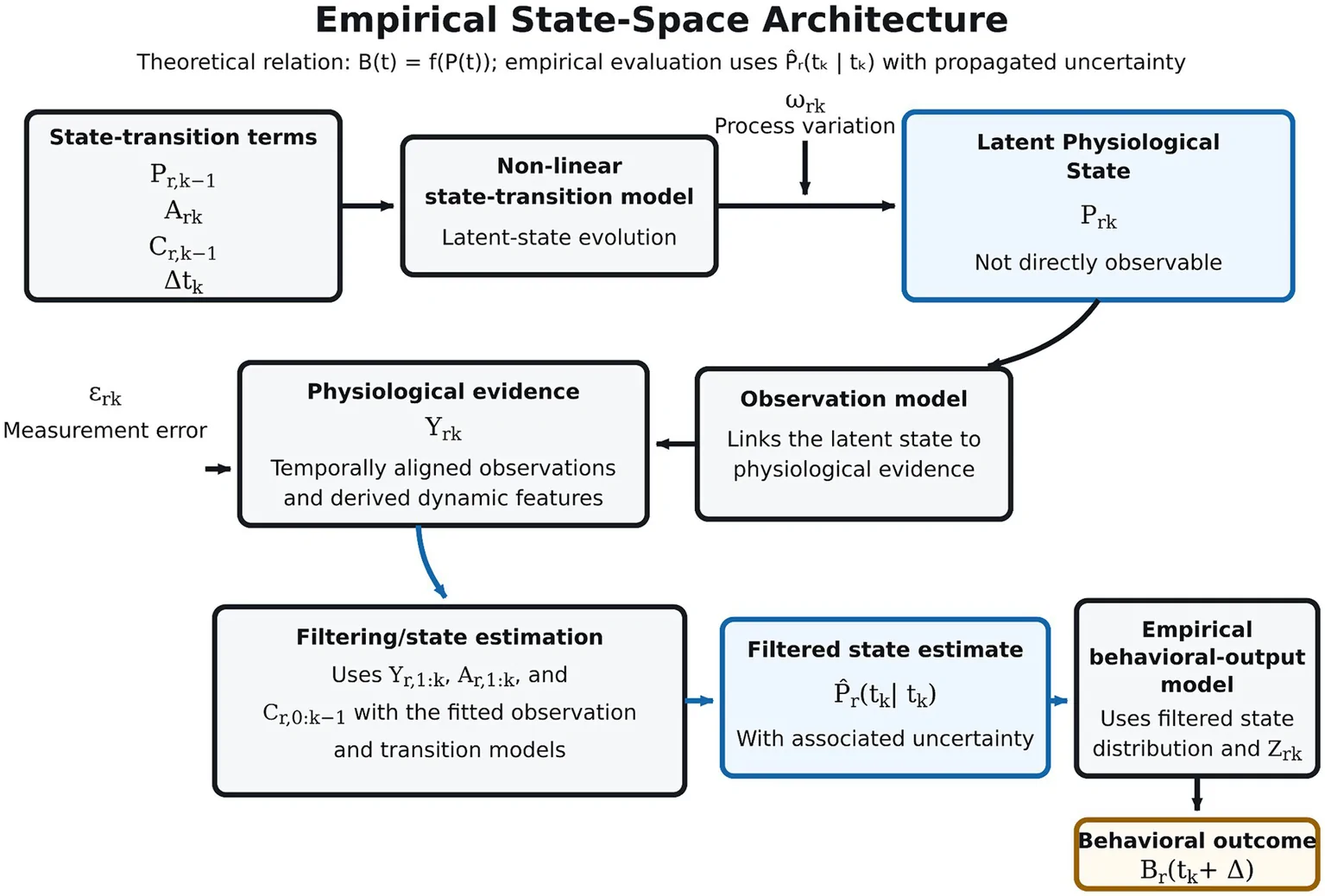
Empirical state-space representation for estimating the physiological state. For participant r at observation k, the preceding latent Physiological State Pᵣ,ₖ₋₁, current measured antecedent conditions Aᵣₖ, relevant prior consequence-related conditions Cᵣ,ₖ₋₁, and elapsed time Δtₖ inform the nonlinear state-transition model describing the evolution of Pᵣₖ, subject to process variation ωᵣₖ. The observation model links Pᵣₖ to the temporally aligned physiological observations and derived dynamic features Yᵣₖ, subject to observation error εᵣₖ. Filtering uses Yᵣ,₁:ₖ, Aᵣ,₁:ₖ, and Cᵣ,₀:ₖ₋₁ with the fitted observation and transition models to obtain P̂ᵣ(tₖ | tₖ) and its associated uncertainty. The behavioral output model uses the filtered state distribution and prespecified comparison variables Zᵣₖ to evaluate the prospective prediction of the separately measured outcome Bᵣ(tₖ + Δ), with state uncertainty propagated. A and relevant prior C inform state evolution but are not elements of Y, whereas B remains outside state estimation. The number and interpretation of latent-state dimensions are study-specific and require theoretical justification, identification, and empirical validation.

## 4 Dimensional structure of P

### 4.1 *P* as a multidimensional state variable

Within the Non-Linear Physiology–Behavior Equation, (P) is the latent, multidimensional, and time-dependent Physiological State generated by ongoing Physiological Organization (Gomez Uncu, 2025b, 2026a). At time (t), it is represented as:


$$P(t)={\left[P₁(t),P₂(t),\ldots ,P_{d}\;(t)\right]}^{T}$$ (6)

Here, (d) is the number of latent-state dimensions supported jointly by the theoretical specification, measurement design, statistical identifiability, and empirical evidence. (Pj​(t)) denotes latent dimension (j) at time (t), and P̂(t) denotes the uncertain empirical estimate of P(t). The state represents an integrated physiological configuration rather than an isolated physiological activity or a simple additive composite.

The framework distinguishes among physiological contributors, functional regulatory domains, dynamic properties and constraints, organizational processes, proposed unifying physiological mechanisms, measurement evidence, and latent-state dimensions. Although related, these categories are not interchangeable. The Thirteen Physiological Systems do not prescribe 13 latent dimensions; the Seven Layers of Physiological State, load, regulatory capacity, variability, coupling, State Constraints, and Physiological Accessibility are not automatically dimensions of (P).

Biological plausibility, statistical estimability, and construct validity provide distinct support for (P). Physiology makes coordinated regulation plausible (Hall, 2016), and state-space methods permit estimation from incomplete, noisy observations (Särkkä and Svensson, 2023), but neither establishes dimensionality or validates its physiological interpretation. Candidate models must therefore be compared for identifiability, stability, residual adequacy, uncertainty, interpretability, out-of-sample performance, parsimony, and reproducibility. Multidimensionality requires a stable, interpretable, reproducible advantage over a one-dimensional state, isolated indicator, established constructs, or a simple composite; equivalent or better simpler models would not support it.

### 4.2 Thirteen physiological systems: contributors to physiological organization. Not dimensions

The Thirteen Physiological Systems organize coverage; they do not imply 13 anatomically discrete systems or latent dimensions. Table 1 summarizes their proposed contributions (Gomez Uncu, 2025a, 2026a). The taxonomy combines conventional organ systems with overlapping sensory, interoceptive, proprioceptive, and metabolic-regulatory domains. Established physiology informs but does not validate this classification (Hall, 2016).

**Table 1:** Thirteen physiological systems and their proposed framework roles.

Physiological system	Proposed framework role
Nervous system (CNS, PNS, AND ANS)	Neural integration, communication, and regulation across internal physiological processes and interactions with external environmental conditions.
Endocrine system	Hormonal signaling involved in stress regulation, metabolism, growth, development, reproduction, and longer-timescale physiological coordination.
Immune system	Inflammatory, defensive, surveillance, restorative, and regulatory processes that interact with neural, endocrine, and metabolic regulation.
Cardiovascular system	Circulation, perfusion, and distribution of oxygen, nutrients, hormones, immune mediators, and metabolic products.
Respiratory system	Gas exchange and respiratory regulation, including interactions with autonomic activity, metabolic demand, and acid–base balance.
Digestive system and gut–brain axis	Digestion, nutrient processing, gastrointestinal signaling, microbiota-related influences, and bidirectional communication with neural, endocrine, immune, and metabolic processes (Mayer et al., 2015).
Reproductive (neuroendocrine–gonadal) system	Reproductive and gonadal regulation and its interactions with endocrine, developmental, and metabolic processes.
Musculoskeletal system	Posture, movement, muscle tone, force production, and the associated metabolic and proprioceptive demands.
Vestibular–proprioceptive system	Orientation, balance, body position, movement detection, and integration of spatial and postural information.
Exteroceptive sensory systems	Physiological detection and processing of visual, auditory, olfactory, gustatory, and other information originating outside the organism.
Tegumentary/tactile–affective system	Barrier regulation, thermoregulation, tactile and nociceptive signaling, and physiological responses associated with bodily contact.
Interoceptive–proprioceptive integrative system	Integration of internal bodily signals with information about body position, movement, and current physiological conditions (Craig, 2009).
Metabolic–mitochondrial/energy-regulation system	Energy production, allocation, and availability, together with metabolic support required for physiological regulation and behavioral activity.

The Thirteen Physiological Systems constitute a framework-specific taxonomy (Gomez Uncu, 2025a, 2026a). Established physiology informs the general descriptions of the systems (Hall, 2016) but does not establish or validate this taxonomy. Sources cited in individual rows support the corresponding physiological processes rather than the framework-specific classification.

Environmental conditions do not become components of (P) merely because they elicit physiological responses. The conditions themselves remain external and are represented as (A) or (C), according to their temporal and functional positions. Only physiological observations or derived features associated with their detection and integration may contribute to Y(t).

Systems are interacting evidence sources, not independent compartments. Network-physiology research supports cross-system coordination but validates neither the taxonomy nor (P) (Bashan et al., 2012). Studies need not measure all 13 Systems but must justify coverage, identify omissions, document overlaps, and distinguish multisystem evidence from multiple features of one signal. Coverage describes measurement breadth, not a score or dimension of (P).

### 4.3 Seven layers as cross-system regulatory domains

The Thirteen Systems organize physiological sources; the Seven Layers organize regulatory functions across them. Table 2 summarizes their roles and boundaries (Gomez Uncu, 2025a, 2026a). Layers are relational, not anatomical: they impose neither sequence, hierarchy, nor seven latent dimensions. Systems and Layers relate many-to-many, so overlapping indicators must not be counted repeatedly. Layers guide selection and interpretation without requiring one measure each. Studies must prespecify each Layer’s target functions and physiological evidence criteria. Within that scope, coverage is full, partial, or absent when all, some, or no target functions meet those criteria. Shared indicators are mapped to relevant Layers but counted once; feature counts and physiologically distinct source counts are reported separately. Studies assessing a subset must limit conclusions accordingly.

**Table 2:** Seven layers of physiological state and their proposed framework roles.

Layer	Proposed framework role and analytical boundary
Environmental–physiology interface layer	Physiological processes through which external environmental conditions interact with the organism and alter regulatory demand. The environmental conditions themselves remain external to P and are represented by A or C.
Sensory processing layer	Physiological detection, transmission, processing, and integration of exteroceptive input. The input remains outside P, whereas its physiological processing occurs within Physiological Organization.
Autonomic layer	Autonomic regulation of readiness, mobilization, stabilization, and recovery through sympathetic, parasympathetic, and related physiological processes.
Interoceptive layer	Detection, transmission, and integration of signals concerning internal physiological conditions (Craig, 2009; Khalsa et al., 2018).
Hormonal–neuroendocrine layer	Hormonal and neuroendocrine regulation across acute, circadian, developmental, reproductive, metabolic, and longer timescales.
Affective/emotional-state layer	Physiological and neuroregulatory organization associated with the formation of affective and emotional states. This Layer is not equivalent to subjective emotion, affect ratings, emotional labels, or observable emotional behavior (Barrett and Simmons, 2015; Seth, 2013).
Cognitive–regulatory layer	Physiological and neuroregulatory organization contributing to the Physiological Accessibility of cognitive Adaptive Capacities, including attention, working memory, inhibitory control, flexibility, and decision-making. These capacities and their task-based manifestation are not physiological indicators of the Layer (Forte et al., 2019).

The Seven Layers of Physiological State are framework-specific functional regulatory domains (Gomez Uncu, 2025a, 2026a). The external sources cited in individual rows support relevant physiological processes but do not establish or validate the Seven-Layer architecture.

Research on interoception, affective regulation, and physiological correlates of cognition supports Layer-relevant processes but not the Seven-Layer architecture (Craig, 2009; Barrett and Simmons, 2015; Seth, 2013; Forte et al., 2019; Khalsa et al., 2018). Only physiological observations and features enter (Y(t)); self-report, affect ratings, cognitive-task performance, and behavior remain external. Layer-informed models require identifiable, reproducible advantages over System-only, isolated-construct, unstructured-indicator, and simple-composite alternatives.

### 4.4 Dynamic properties linking physiological organization to accessibility

Physiological Load, Regulatory Capacity, State Constraints, and Physiological Accessibility are distinct. Load denotes current and accumulated regulatory demands. Regulatory Capacity is the time-dependent ability to allocate resources, coordinate responses, buffer perturbations, sustain organization, and recover. State Constraints may limit state evolution or availability of existing Adaptive Capacities. Physiological Accessibility is their state-dependent availability for recruitment and use. None is automatically a System, Layer, indicator, or latent dimension of P (Gomez Uncu, 2025a, 2026a).

Physiological Load differs from the external demands (A) or (C) and allostatic load, the cumulative physiological costs of repeated or insufficiently regulated adaptation (McEwen, 2000; Sterling, 2012). Elevated Load is not inherently pathological. Load and Regulatory Capacity are interacting dynamic properties, not independently observed quantities or a fixed ratio.

The proposed state-dependent pathway requiring prospective testing is: Load and Regulatory Capacity condition Physiological Organization; Physiological Organization generates (P(t)); and associated State Constraints influence Physiological Accessibility. Regulatory Capacity differs from an Adaptive Capacity. Restricted Accessibility means an existing capacity may be temporarily unavailable, not lost. Over longer periods, (P) trajectories may shape acquisition, strengthening, retention, and generalization of new Adaptive Capacities. That developmental process differs from momentary accessibility and is not determined by (P) alone.

Operationalizing requires prespecified physiological evidence, windows, person-specific reference, uncertainty, and comparison models. Behavior alone cannot establish these constructs. Testing Physiological Accessibility requires independent confirmation of the Adaptive Capacity, estimation of P̂(t) before the target behavior, and exclusion of B(t + Δ) from Y(t).

### 4.5 Variability as evidence, not state

Variability occurs in observed physiology Y(t), estimated latent-state P̂(t), and behavior B(t + Δ); these levels are not interchangeable. Physiological variability may inform estimation of (P) but is not automatically noise, a state dimension, or evidence of adaptation or dysfunction. Behavioral variability cannot establish change in (P) or Physiological Accessibility.

Candidate features include baseline, successive differences, reactivity, persistence, recovery, entropy, complexity, and frequency-specific variability. Interpretation depends on the source signal, System, condition, and timescale, and may vary with the window, sampling, activity, posture, respiration, medication, rhythms, artifacts, missingness, and preprocessing (Goldberger et al., 2002; Kelso, 1995). Features require theoretical justification, reliability at the intended timescale, and prospective comparison with simpler alternatives.

Person-specified baselines and dynamic features must use prespecified procedures and windows containing only observations available by prediction time t.

### 4.6 Cross-system integration and coupling

The framework treats Cross-System Integration and Coupling as a time-varying property of Physiological Organization. It refers to relationships among physiological processes that may change with Physiological Load, Regulatory Capacity, prior-state history, environmental demands, and ongoing physiological reorganization. It is not equivalent to (P), an additional System or Layer, or a latent dimension specified in advance.

For two temporally aligned physiological signals Yₘ and Yₙ, a pairwise coupling feature may be defined as:


$$\kappa_{\mathrm{mn}}(t;w)=M_{\mathrm{mn}}(Y_{m}[W(t,w)],Y_{n}[W(t,w)])$$ (7)

In Equation 7, W(t,w) = (t − w, t] is the prespecified observation window of width w ending at t in prospective analysis; Mₘₙ is the selected estimator of the relationship between signals m and n; and κₘₙ(t;w) is the resulting coupling feature. κₘₙ(t; w) may be scalar- or vector-valued; its dimension and component indexing, including any lag or frequency selection, must be prespecified. Depending on the signals and hypothesis, Mₘₙ may estimate a lagged association, spectral coherence, phase synchronization, mutual information, network connectivity, or another theoretically justified relationship. The symbol κ distinguishes these coupling features from the matrix (K) introduced in Equation 11.

A coupling feature may enter Y(t) as derived physiological evidence only when its construction is fully prespecified. The protocol must identify the source signals, synchronization procedure, preprocessing steps, observation window, estimator, expected relationships, uncertainty, and statistical decision rule. This specification is necessary because apparent coupling may arise from common environmental input, shared physiological drivers, movement, respiration, sensor artifacts, autocorrelation, preprocessing choices, or measurement error. Neither stronger nor weaker coupling has a universal interpretation: greater coupling is not necessarily adaptive, and reduced coupling is not necessarily dysfunctional.

Coupling evidence cannot independently establish continuous physiological integration or a particular unifying physiological mechanism. Allostatic Load Redistribution is addressed in Section 6.6 as a proposed unifying physiological mechanism. Its evaluation requires a prespecified, temporally ordered differential pattern across relevant Systems or Layers that performs better than uniform-activation, common-input, independent-system, and measurement-artifact alternatives.

### 4.7 Integrated multidimensional representation

The framework separates the physiological state from the concepts and evidence used to characterize it. Physiological Organization is the continuous process that generates P(t), whereas P̂(t) is a model-based estimate of that momentary state from partial and fallible physiological evidence. The 13 Systems and Seven Layers organize measurement coverage and interpretation; dynamic properties describe how a state may change; organizational processes specify broad relationships within the framework; and unifying physiological mechanisms propose operations that may generate observed patterns. None of these categories automatically defines the coordinates of (P).

A multidimensional representation is supported only if its dimensions are statistically identifiable, accompanied by appropriate uncertainty, physiologically interpretable, stable across reasonable analytical choices, and prospectively informative beyond lower-dimensional and simpler alternatives. Failure to satisfy these criteria weighs against the tested representation. It does not justify retrospective relabeling of dimensions, redefining states, or selecting a more favorable interpretation after observing the behavioral results.

## 5 Indicator and measurement architecture for estimating *P*

Operationalizing (P) requires distinguishing the unobservable state from its evidence. Generated by Physiological Organization, (P) is not an indicator, feature, regulatory construct, or behavioral outcome (Gomez Uncu, 2025a, 2026a). Its empirical counterpart, P̂(t), is a model-dependent estimate from aligned, incomplete physiological evidence.

Estimating (P) is a partial-observability problem. Equation 3 relates (Y(t)) to (P(t)); Equation 5 models (P) from its preceding state, (A), prior (C), elapsed time, and process variation. State-space methods accommodate incomplete, noisy observations (Kalman, 1960; Särkkä and Svensson, 2023), but do not establish (P), its dimensions, or interpretation.

The architecture assigns noninterchangeable roles:

- Physiological evidence: Physiological observations and derived physiological features constitute Y(t).
- External conditions: (A) and (C) remain separately measured environmental variables that may enter the state-transition model.
- Behavioral outcome and external validation: (B) remains outside (Y(t)) and serves as a downstream outcome or external validation variable. Self-reports, affect ratings, cognitive-task performance, and diagnostic classifications likewise remain outside the physiological observation vector.

For prospective B(t + Δ), neither the outcome nor post-(t) information may contribute to P̂(t).

Table 3 summarizes development and evaluation of P̂(t). Studies need not cover all Thirteen Systems or Seven Layers but must justify coverage, overlaps, and gaps. P̂ is population-, context-, timescale-, observations-, processing-, and model-dependent, not universal. Unidentifiable or unstable estimates, or no reproducible value beyond environmental variables, prior behavior, indicators, established constructs, and composites, count against the operationalization.

**Table 3:** Operational sequence for developing and evaluating P̂(t).

Step	Required specification
1. Define the target and temporal structure	Prespecify B, the unit of analysis, physiological and behavioral timescales, the state-estimation time and observation windows, the prediction interval Δ, and the temporal positions of A and relevant prior C.
2. Specify coverage and select evidence	Identify the physiological processes, Systems, Layers, and dynamic properties relevant to the hypothesis. Select observations according to theoretical relevance, reliability, sensitivity, temporal resolution, feasibility, artifact susceptibility, and nonredundancy.
3. Acquire, preprocess, and align	Prespecify recording conditions, sampling and synchronization procedures, quality-control and artifact procedures, transformations, missing-data handling, person-specific baselines, modality-specific delays, and temporal alignment.
4. Derive features and specify models	Prespecify feature definitions and windows, observation-to-state relationships, dimensionality, identification constraints, loadings or weights, shared measurement sources, and error structure. Treat equal weighting as a comparator rather than the default integrating method.
5. Estimate P̂ and uncertainty	Select an estimator appropriate to the specified model and available data; Distinguish process variation from observation error and report state and parameter uncertainty. Use filtered estimates for prospective prediction and smoothed estimates only for retrospective reconstruction.
6. Evaluate validity and comparative performance	Evaluate identifiability, reliability, temporal coherence, convergent and discriminant evidence, prospective and incremental validity, calibration, robustness, and reproducibility in appropriately separated data and against prespecified simpler alternatives.

These steps define the methodological sequence for developing and evaluating an empirical operationalization of P̂(t). They are not Regulatory Stages, Organizational Processes, or Unifying Physiological Mechanisms.

### 5.1 Observable physiological indicators and derived features

An observation is measured directly in Y(t); a feature is computed within a prespecified window. For example, an electrocardiographic signal is an observation, whereas derived heart rate variability is a feature. Sources and derivatives are not automatically independent evidence for (P).

Candidates include cardiovascular, respiratory, electrodermal, temperature, neurophysiological, vestibular, proprioceptive, interoceptive, and sensory-related observations; endocrine, immune, metabolic, musculoskeletal, sleep, and circadian measures; none is universally required. Heart rate variability informs assessment of cardiac autonomic modulation but is not Regulatory Capacity or (P) (Task Force of the European Society of Cardiology and the North American Society of Pacing and Electrophysiology, 1996; Thayer et al., 2012). Electrodermal activity reflects sympathetic sudomotor processes, not complete arousal, emotion, stress, or (P) (Boucsein et al., 2012). Intermittently measured biomarkers require timing models; accelerometry may identify activity or artifacts but is not inherently physiological evidence.

Selection should reflect theory, reliability, within-person sensitivity, resolution, feasibility, artifact susceptibility, and nonredundancy—not (B) alone. Report acquisition units, sampling, preprocessing, quality-control, person-specific baselines, missingness, and shared sources. Relevant behavioral, biological, environmental, sensor, and assay influences must be controlled, measured, or modeled (Cacioppo et al., 2017; Hall, 2016). A small, coherent set may outperform noisy, overlapping measures. Behavioral measures remain outside Y(t).

### 5.2 Temporal windows, state estimation, and regulatory stage

*Momentary* denotes time (t), not the duration of the evidence used. P(t) is the latent state at (t), whereas P̂(t) is its uncertain model-based estimate, typically based on a finite window. An ordered sequence forms a trajectory. A state transition is evolution between observation times; *a regime transition* is movement between prespecified distinguishable regions. A Regulatory Stage describes a proposed period or process within Physiological Organization. Neither of the latter two can be inferred from an isolated signal or behavioral change.

For modality (j), let (R_j_(s)) denote the raw or preprocessed physiological samples observed over time (s). At state-estimation time tₖ, the modality-specific observation window and extracted feature are defined jointly as:


$$W_{\mathrm{jk}}=[t_{k}-\ell_{j}-w_{j},t_{k}-\ell_{j}]$$

$$Y_{j}(t_{k})=\varphi_{j}(\{R_{j}(s):s\in W_{\mathrm{jk}}\})$$ (8)

where (w_j_ > 0) is the window width, (ℓ_j_ ≥ 0) a prespecified offset, φ_j_ the feature-extraction function, and Y_j_(tₖ) is a component of Y(tₖ). The nonnegative offset prevents future data use. Windows may differ because continuous cardiovascular, respiratory, electrodermal, and neurophysiological measurements and intermittent endocrine, immune, and metabolic measurements have different timescales. Assay and physiological-process timing must be distinguished. Interpolation and last-observation-carried-forward alter representation but create no information; elapsed time, missingness, and uncertainty must represent irregular sampling.

A filtered estimate (P̂(tₖ | tₖ)) uses permitted information through (tₖ); a smoothed estimate (P̂(tₖ | T)), (T > tₖ), uses later information and is retrospective (Särkkä and Svensson, 2023). Prospective (B(tₖ + Δ)) prediction requires windows ending by (tₖ) and a positive, justified, prespecified Δ. Future physical observations, future (A) or (C) values, and contemporaneous or future (B) are excluded.

Table 4 summarizes the Seven Stages of Temporal Regulation and proposed boundaries (Gomez Uncu, 2025a). Stages need not have universal durations, be exclusive or sequential, or represent distinct latent states. Classification requires independent or prespecified features, reference conditions, duration criteria, rules, and uncertainty thresholds, plus comparison with continuous, nonstage, and nonswitching alternatives (Kelso, 1995; Strogatz, 2015).

**Table 4:** Seven stages of temporal regulation and their operational boundaries.

Stage	Proposed temporal pattern and boundary
Baseline regulatory stability	Relative physiological coordination used as a person-specific reference. It does not imply an absence of variability, an optimal state, or conformity to a population norm.
Input and regulatory demand	A period during which environmental conditions represented by A or relevant prior C interact with P and contribute to changing physiological regulatory demands. The conditions, timing, and required physiological evidence must be specified.
Threshold activation	A proposed point or region at which interacting demands are associated with a nonproportional change in the organization or trajectory of P; it cannot be established through visual inspection or an arbitrary cutoff in a single indicator.
Regulatory buffering	Physiological adjustment through which coordination may be maintained temporarily despite continuing demand. It cannot be inferred solely from behavioral stability or the absence of observable change.
Regulatory destabilization	A sustained or reproducible departure from reference dynamics in persistence, variability, coupling, recovery, or another prespecified feature that is distinguishable from transient reactivity, artifacts, and uncertainty.
Recovery processes	Physiological-regulatory processes following a defined perturbation through which demand may decrease, and organization may stabilize or change. Recovery does not necessarily entail an immediate or complete return to baseline.
State reorganization	Persistence within, or movement toward, a different physiological configuration following regulatory change. Reorganization does not necessarily indicate improvement, deterioration, or a return to baseline.

The Seven Stages of Temporal Regulation are framework-specific classifications (Gomez Uncu, 2025a). They are not fixed-duration, universal, mutually exclusive, or strictly sequential periods, nor do they automatically constitute distinct Physiological States. These boundaries are conceptual; empirical applications must prespecify whether classification uses deterministic rules, probabilistic assignments, or inferred latent regimes and validate the chosen approach.

### 5.3 Component-wise measurement and transition models

Equation 6 defines (P(t)) as a ( d )-dimensional vector, represented as $\hat{P}(t)={\left[\hat{P}₁(t),\hat{P}₂(t),\ldots ,\hat{P}d(t)\right]}^{T}$ . Its dimensions are model-based coordinates, not automatic representations of System, Layers, indicators, Physiological Load, Regulatory Capacity, Variability, or coupling.

For (q) physiological observations or derived features, the component-wise observation model is:


$$Y_{j}(t_{k})=h_{j}(P(t_{k});\theta_{j})+\varepsilon_{\mathrm{jk}},\;\;j=1,\ldots ,q$$ (9)

where (h_j_) maps complete P(tₖ) to expected (Y_j_(tₖ)), (θ_j_) contains measurement parameters; and (ε_jk_) represents error and residual variation. Observations may inform several dimensions and one dimension may be estimated from several observations, but such cross-loadings require theoretical justification and identification constraints.

The corresponding component-wise transition model is:


$$P_{i}(t_{k})=g_{i}(P(t_{k-₁}),A(t_{k}),C(t_{k-1}),\Delta t_{k};\theta_{i})+\omega_{\mathrm{ik}},i=1,\ldots ,d$$ (10)

In Equation 10, ( $g_{i}$ ) models dimension (i) from the preceding state and permitted inputs; (ωᵢₖ) represents unexplained process variation. Equation 9 addresses measurement; Equation 10 addresses evolution. Indicator associations do not establish latent interactions, and cross-dimensional effects do not imply biological Systems. Nonlinearity in observation ( $h_{j}$ ) and transition ( $g_{i}$ ) functions must be tested separately.

( d ) is not fixed at 13, seven, or ( q ). Compare theory-constrained dimensions using identifiability, convergence, residual adequacy, uncertainty, stability, out-of-sample performance, parsimony, and reproducibility. Report scale, sign, ordering, and rotation constraints; model dependence arising from shared signals; ensure measurement comparability; and retain uncertainty reflecting quality and availability.

### 5.4 Candidate empirically estimable representation

Equations 1, 2 express theoretical dependencies; (F) and (f) are structural placeholders, not estimators. Equations 3, 5, 9, and 10 provide a general observation-transition architecture. The following specification is one candidate implementation, not a unique or validated representation of (P) (Auger-Méthé et al., 2021; Särkkä and Svensson, 2023).

For individual (r), at regularly spaced times (k = 1, …, n), a candidate transition model is:


$$\begin{array}{l} P_{\mathrm{rk}}=\alpha +\Phi P_{r}{,}_{k-1}+\Gamma \_A\;A_{\mathrm{rk}}+\Gamma \_C\;C_{r}{,}_{k-1}\\ +K(P_{r}{,}_{k-1}⊗P_{r}{,}_{k-1})\\ +\mathrm{\Psi A}(A_{\mathrm{rk}}⊗P_{r}{,}_{k-1})\\ +\mathrm{\Psi C}\;(C_{r}{,}_{k-1}⊗P_{r}{,}_{k-1})\\ +H{\left[\zeta (P_{r}{,}_{k-1};\theta \_\zeta )-\tau \right]}_{+}+u_{r}+\omega_{\mathrm{rk}}. \end{array}$$ (11)

(α) contains (d) intercepts, and (Φ) represents persistence and cross-dimensional carryover. (Γ_A and Γ_C) map measured (A) and (C) inputs into the subsequent (d)-dimensional state. (K) maps preceding state products; (Ψ_A) and (Ψ_C) map environment-state interactions. Duplicate symmetric Kronecker products require removal or constraints. (uᵣ) is a mean-zero person-specific transition effect constant across occasions; its distribution, covariance, and dependence on the initial state and inputs must be prespecified; (ωᵣₖ) is time-specific process variation. Thus, comparable conditions may yield different trajectories depending on prior state, without implying that (P) changes the environmental event.

(ζ) defines the threshold index, (τ) the candidate boundary, and (H) maps positive threshold excess into the state vector; and ${\left[x\right]}_{+}=\max(x,0)$ . A fitted cutoff does not establish Threshold Activation, a distinct State, or a Multisystem Threshold Effect. Such claims require prespecified criteria, aligned multisystem evidence, replication, and linear, smooth-nonlinear, and nonthreshold comparisons. Any threshold selection must use model-development data, with the specification and comparison rules fixed before confirmatory evaluation. Equation 11 need not contain every term; additions require theory, identifiability, and sufficient repeated observations. Irregular timing requires Δt-dependent parameters, continuous-time modeling, or another justified method. (B) remains outside the transition model.

One candidate nonlinear physiological observation model is:


$$Y_{\mathrm{rk}}=h(P_{\mathrm{rk}};\theta_{Y})+\varepsilon_{\mathrm{rk}}$$ (12)

With the following linear comparison model:


$$Y_{\mathrm{rk}}=\mu +\Lambda P_{\mathrm{rk}}+\varepsilon_{\mathrm{rk}}$$ (13)

where (h) maps (P) to expected physiological observations, μ is a (q)-dimensional intercept, Λ is a q × d loading matrix, and εᵣₖ is the observation error. Indicator relationships, cross-loadings, scale constraints, reliability, stability, and correlated residuals require specification. Implementations must prespecify identifying constraints for the chosen models. Comparisons across individuals or contexts require evaluation of measurement invariance and justification of common latent coordinates. Process variation and observation error remain distinct; (A) and (C) remain outside Y, and (B) remains outside the physiological observation model. Equations 12, 13 assume no direct A or C effects on physiological observations conditional on P; these measurement assumptions require empirical evaluation.

Let Zᵣₖ contain prespecified environmental, historical, design, and comparison variables. One behavioral-output model is:


$$E[B_{r}(t_{k}+\Delta )\;|\;P_{\mathrm{rk}},Z_{\mathrm{rk}}]$$

$$=\ell_{B^{-1}}\;(\beta_{0}+\beta^{T}P_{\mathrm{rk}}+{P_{\mathrm{rk}}}^{T}\Omega P_{\mathrm{rk}}+\gamma^{T}Z_{\mathrm{rk}})$$ (14)

In Equation 14, β represents linear state effects, (Ω) quadratic and cross-dimensional effects, γ associations with (Z), and (ℓ_B^−1^) the outcome’s inverse link. Only (Ω)‘s symmetric component is identifiable, so it requires a symmetry constraint or unique quadratic and cross-product terms. Compare this specification with linear-only state terms. Including (Z) adjusts the outcome model without incorporating (Z) into (P), permitting evaluation of (P)‘s incremental contribution.

Prospective evaluation requires filtered estimates of P(t) that exclude target (B), separate model development and confirmatory testing, and propagated state and prediction uncertainty. Maximum-likelihood, Bayesian, nonlinear Gaussian, particle, or hierarchical estimators may be used when appropriate. Comparisons must include (A) and (C) without (P), prior behavior, isolated indicators, established stress or arousal constructs, allostatic-load indices, composites, lower-dimensional states, linear transitions, and nonthreshold dynamics. Unidentified estimates or no reproducible prospective improvement would count against the operationalization.

### 5.5 Illustrative prospective implementation

Consider a prospective repeated-measures study of initiation latency for a standardized, mastered task, for example, placing a familiar object in a container after “Begin.” Before data collection, define the Adaptive Capacity, mastery criterion, initiation response, and latency interval, and demonstrate capacity independently of the target trials. This distinguishes state-dependent Physiological Accessibility from incomplete learning or absence of capacity. Mastery must be verified in separate pretest trials, with a prespecified validity period and reassessment criteria.

For participant (r), (t_k) ends the preinstructional physiological window and marks instruction delivery on trial (k). (A_rk) includes the instruction, difficulty, materials, sensory features, transition, and setting—each standardized or measured. Prior (C_r,k-1) may include feedback, assistance, reinforcement, and breaks. Record motivating operations, trial order, prior latency, intertrial interval, exposure, and carryover.

Physiological evidence (Y_rk) may include electrocardiographic signals and derived heart rate or heart rate variability, respiration, electrodermal activity, and peripheral temperature. Each modality uses a prespecified window ending at (t_k). Same-source features are not automatically independent. Equations 11–13 define candidate transition and observation models. One filtered estimate is:


$${\hat{P}}_{r}(t_{k}\;|\;t_{k})=E[P_{\mathrm{rk}}\;|\;Y_{r},₁{:}_{k},A_{r,1:k},C_{r,0:k-1}]$$ (15)

Equation 15 is the filtered distribution’s conditional mean, not a universal or error-free estimator. Although (A) and prior (C) inform state evolution, they remain outside (Y_rk), and post-( $t_{k}$ ) information is excluded. Differences between conditional means establish distinct states only when uncertainty and a prespecified separation criterion support them.

Let L_rk be the interval from the instruction to the first qualifying response. Latency belongs to (B), outside Y_rk. For positive, uncensored latencies, one candidate model is:


$$\log\;L_{\mathrm{rk}}=\beta_{0}+\beta^{T}P_{\mathrm{rk}}+{P_{\mathrm{rk}}}^{T}\Omega P_{\mathrm{rk}}+\gamma^{T}Z_{\mathrm{rk}}+b_{r}+\xi_{\mathrm{rk}}$$ (16)

Here, Z_rk contains comparison variables, b_r is a distributed, mean-zero participant-specific effect with a specified distribution, and $\xi_{\mathrm{rk}}$ is the residual variation. Ω requires Equation 14’s symmetry or unique-term constraint. Equation 16 requires supported log-latency assumptions. Noninitiation within the prespecified observation interval produces right-censored data and requires a time-to-event model, not an arbitrary maximum latency.

Predictions must integrate over the filtered distribution of $P_{\mathrm{rk}}$ rather than treat its mean as error-free. Across trials comparable in (A), prior (C), motivating operations, task conditions, learning history, and demonstrated capacity, prespecified state-distribution differences should predict reproducible conditional-latency differences after adjustment for (Z). Direction, contrast, and minimum meaningful difference must be prespecified or learned in training data and tested on held-out observations. Prediction is probabilistic and not necessarily linear.

Support requires identifiable filtered states, prediction, incremental value beyond alternative models, and explicit uncertainty. Such evidence supports Physiological Accessibility without directly measuring it or establishing mediation. Measurement failure weighs against estimation; predictive failure against P’s contribution; and nonlinear failure against terms alone.

## 6 System dynamics and nonlinearity

### 6.1 Distinct sources of coupling and nonlinearity

The framework proposes time-dependent coupling between (A) and (P), and between (P) and (C), while (P) influences Physiological Accessibility and thereby constrains, but does not uniquely determine, the downstream behavioral outcome (B) (Gomez Uncu, 2025b, 2026a). Equation 1 models change in (P) as conditional on (A), current (P), and relevant (C). It does not model the evolution of (A) or (C) and therefore cannot, by itself, establish reciprocal causation. Environmental consequences following (B) may enter later intervals as (C) and, when temporally and functionally appropriate, as later (A), without placing (B) inside the A↔ P ↔ C relation. Testing bidirectional coupling requires separately specified models of how A and C evolve in relation to prior P.

Organism–environment coupling must be distinguished from Cross-System Integration and Coupling within Physiological Organization. In the first relation, (A) and (C) are external conditions whose modeled contributions may depend on the preceding physiological state. In the second, interactions among Systems and Layers are internal organizational relations from which (P) is generated. Physiological mediation is proposed by the framework but is not established by these relations alone.

The candidate transition model in Equation 11 locates nonlinearity in state-by-state products, (A)-by-(P) interactions, (C)-by-(P) interactions, and threshold terms. These terms must be distinguished from two other possible sources of nonlinearity: the observation function may relate (P) nonlinearly to (Y), and the behavioral-output function may relate (P) nonlinearly to (B). Evidence of nonlinearity at one location does not establish nonlinearity at another. In particular, apparent nonlinear structure in (Y) may arise from measurement characteristics, artifacts, or preprocessing rather than state evolution.

These dynamics form part of Nonlinear Adaptive Dynamics, an organizational process in Table 5. Here, *Adaptive* refers to demand-sensitive physiological adjustment, not to an inherently beneficial, optimal, or successful outcome. A small demand may produce a large state change, whereas a great demand may produce a small change; the response may depend on preceding (P), Physiological Load, Regulatory Capacity, recovery history, cross-system organization, and proximity to a candidate threshold region (Gomez Uncu, 2025a, 2026a). State-interaction, smooth-nonlinear, quadratic, and threshold models must be compared with additive linear alternatives using analytically separated data. If the additional terms do not improve reproducible prediction, the evidence weighs against those terms. It does not justify retrospectively attributing unexplained variation to an unspecified form of nonlinearity (Kelso, 1995; McEwen, 2000; Sterling, 2012).

**Table 5:** Organizational Processes and Unifying Physiological Mechanisms.

Classification	Construct	Framework role	Empirical requirement and condition of non-support
Organizational process	Continuous physiological integration	Ongoing coordination among the Thirteen Physiological Systems across the Seven Layers through which Physiological Organization is maintained and reorganized.	Requires reproducible, temporally aligned evidence of coordination across physiologically distinct systems that cannot be explained adequately by common environmental input, independent signal changes, shared physiological drivers, measured error, or artifacts. Evidence from one indicator or an isolated statistical association is insufficient.
Organizational process	State-dependent accessibility	Process through which P, via its associated State Constraints, influences the Physiological Accessibility of existing Adaptive Capacities.	Requires independent evidence that the relevant capacity exists, a filtered estimate P̂(t) based only on information available through time t, and a separately measured prospective behavioral outcome B(t + Δ). Behavioral change alone cannot establish Physiological Accessibility, and an association between P̂ and B does not establish mediation.
Organizational process	Nonlinear adaptive dynamics	Evolution of Physiological Organization and successive states across time and changing conditions through prior-state dependence, interaction, buffering, transition, destabilization, recovery, and reorganization.	Requires prespecified nonlinear or state-dependent terms to improve performance in separated data beyond appropriate linear, additive, no-history, nonthreshold, and nonswitching alternatives. If simpler alternatives perform equally well or better, the proposed nonlinear specification is not supported.
Unifying physiological mechanism	Predictive interoceptive coding	Proposed operation through which physiological history, bodily and sensory information, learned environmental cues, and anticipated demands contribute to anticipatory regulation and subsequent updating.	Requires a prespecified physiological response that precedes the realized demand and evidence of predicted updating when anticipated and realized conditions differ. The mechanism is not supported if the pattern is explained adequately by autocorrelation, concurrent sensory input, direct cue-response associations, movement preparation, or other specified alternatives without evidence of anticipatory updating (Barrett and Simmons, 2015; Seth, 2013).
Unifying physiological mechanism	Allostatic load redistribution	Proposed reorganization of Physiological Load and regulatory activity across interacting Systems and Layers as demands change; redistribution is not defined as a fixed quantity or necessarily as a zero-sum transfer.	Requires a prespecified temporally ordered differential pattern across relevant Systems or Layers that is distinguishable from uniform activation, common input, independent system change, shared artifacts, and simple composite explanations (McEwen, 2000; Sterling, 2012).
Unifying physiological mechanism	Multisystem threshold effects	Proposed contribution of accumulated and interacting demands across multiple Systems and Layers to threshold-dependent reorganization.	Requires converging, temporally aligned multisystem evidence and reproducible improvement over linear transition, smooth nonlinear, and nonswitching alternatives. An isolated physiological discontinuity or behavioral change is insufficient.

Organizational Processes and Unifying Physiological Mechanisms are distinct framework classifications. A mechanism may contribute to one or more Organizational Processes, but no one-to-one correspondence is assumed. The classifications and construct names derive from Gomez Uncu (2025a, 2026a); Predictive Interoceptive Coding and the three Organizational Processes are further specified in Gomez Uncu (2026a). Sources cited in individual rows support the general plausibility of the relevant processes but do not establish or validate the framework-specific mechanisms.

### 6.2 State evolution, regime transitions, and multisystem threshold effects

The trajectory of (P) may change gradually or exhibit a rapid, disproportionate movement within or between regions of the state space. Nonlinearity does not require an abrupt change during every observation interval. Three concepts must therefore remain distinct. A candidate threshold is a prespecified or estimated boundary or region at which the transition relation changes nonproportionally. A threshold-associated regime transition is an empirically supported change in the trajectory or regime of (P) occurring in relation to that boundary. A Multisystem Threshold Effect is the proposed physiological mechanism through which interacting demands and organization across multiple Systems and Layers contribute to threshold-dependent reorganization (Gomez Uncu, 2025a, 2026a).

A statistical change point does not, by itself, establish a transition in (P); likewise, an estimated transition in (P) does not establish a Multisystem Threshold Effect. In Equation 11, the hinge term permits the slope of the state-transition function to change when the state-dependent index exceeds the candidate boundary (τ). Because the hinge function is continuous at (τ), this term does not require a discontinuity and does not directly measure the proposed physiological mechanism. Evidence for a Multisystem Threshold Effect requires temporally aligned observations from multiple physiological systems or a validated cross-system feature; a prespecified threshold function; a prespecified criterion stating the minimum elapsed time, observation count, or posterior probability required; uncertainty estimates for threshold boundary and transition time; temporal replication; and robustness to artifacts and relevant covariates. When the threshold is derived empirically, it must be evaluated in held-out observations.

Threshold models must outperform otherwise comparable linear-transition, smooth nonlinear, and nonswitching alternatives. Neither a discontinuity in a single physiological signal nor a behavior change is sufficient evidence, and (B) must remain outside (Y). Failure to reproduce the threshold-associated pattern, demonstrate multisystem support, improve prediction in separated data, or exclude artifacts and simpler explanations would count against the proposed mechanism. Moreover, a detected transition is not inherently adaptive or dysfunctional; its interpretation depends on the preceding state, Systems and Layers involved, the relevant demands, recovery dynamics, and subsequent physiological reorganization (Hamilton, 1989; Killick et al., 2012; Kelso, 1995).

### 6.3 Prior-state dependence and historical memory

The subsequent trajectory of (P) may depend on both its immediately preceding state and the history through which that state developed. Equation 11 represents first-order dependence through (ΦP_r,k-1) and allows additional dependence through state–state, environment-by-state, and threshold terms. These terms quantify statistical dependencies within the transition model; they do not, without additional evidence, identify biological mechanisms or causal pathways.

State persistence, carryover, and longer-term historical dependence require separate definitions. Persistence is dependence on the immediately preceding state. Carryover is the continued influence of earlier conditions after those conditions have changed or ceased. Longer-term dependence is supported only when information from earlier intervals improves prediction beyond the immediately preceding state and the other components of the current transition model. Longer-term dependence may be represented through prespecified additional lags, cumulative Physiological Load features, time-since-perturbation, recovery duration, or an augmented state representation. Environmental history remains represented through (A) or (C), whereas behavioral history remains represented through prior (B) or separate covariates. Neither environmental nor behavioral history automatically becomes part of (P).

Prior-state dependence must be evaluated prospectively using filtered state estimates and compared with no-history, first-order, longer-memory, linear, and nonlinear alternatives. Apparent historical dependence may instead reflect temporal trends, biological rhythms, autocorrelated artifacts, correlated measurement error, activity, posture, incomplete measurement of (A) or (C), prior behavior, or learning history. The framework does not presume hysteresis, chaos, or mathematical sensitivity to initial conditions, whereby arbitrarily small differences in initial conditions generate increasingly divergent trajectories (Strogatz, 2015). If preceding-state terms are unstable or fail to improve held-out prediction, the tested form of prior-state dependence would not be supported. Conversely, superior performance by a longer-memory model would challenge the first-order approximation without necessarily refuting historical dependence more broadly.

### 6.4 Persistence, stability, recovery, and variability

Persistence, local stability, instability, and recovery describe related but distinct properties of an estimated state trajectory. Persistence within a region is the tendency of (P) to remain within a defined state-space region. Local stability is the tendency of (P) to return toward a defined reference region following a perturbation. Instability is indicated by reduced persistence, increasing departure from a reference region, or amplification of perturbations. Recovery is the stabilization or reorganization of (P) following a defined change in demand. Prespecified event markers must identify perturbation onset and offset; prespecified physiological and duration criteria must identify recovery onset and completion. Each property must be evaluated from the estimated trajectory and its uncertainty rather than inferred from an isolated physiological signal.

A reference region must be defined using a person-specific baseline, a theoretically specified configuration, or an empirically supported regime with explicit boundaries and temporal criteria. Stability does not imply an absence of variability, physiological health, optimal functioning, high Regulatory Capacity, or unrestricted Physiological Accessibility. Similarly, instability may accompany adaptive transition, Recovery Processes, or broader state reorganization and therefore is not inherently dysfunctional (Gomez Uncu, 2025a, 2026a).

Variability in Physiological observations (Y), variability in the estimated latent-state P̂, and variability in behavior (B) must remain analytically distinct. Candidate measures include persistence, dwell time, transition probability, distance from a reference region, return rate, recovery slope or duration, recurrence, entropy or complexity, and time-varying coupling. Their interpretation depends on the signal, state region, perturbation, person-specific baseline, sampling interval, timescale, and environmental conditions. Accordingly, greater interval variability or stronger coupling should not be interpreted as universally beneficial. Attractor-like organization may be investigated using trajectories sufficiently long and densely sampled to distinguish reproducible convergence, recurrence, or return from autocorrelation and periodic rhythms. It should not be inferred from visual coherence in a plotted trajectory alone (Goldberger et al., 2002; Kelso, 1995; Strogatz, 2015).

Analyses must account for measurement error, artifacts, missing data, movement, posture, activity, medication, illness, sleep, physiological rhythms, preprocessing decisions, and changes in environmental conditions. Support requires prespecified dynamic patterns that exceed state-estimation uncertainty, replicate across appropriate observations or samples, and provide prospective information about subsequent physiological observations, state evolution, or separately measured (B) beyond stationary, trend-based, linear, and other simpler models. Failure to observe a prespecified pattern within the observation interval establishes only that the pattern was not detected during that interval; it does not justify retrospectively reclassifying the interval as another Stage or Organizational process.

### 6.5 Organizational processes governing state and accessibility

The framework identifies three Organizational Processes: Continuous Physiological Integration, State-Dependent Accessibility, and NonLinear Adaptive Dynamics. These processes describe the broad relationships through which Physiological Organization is maintained and reorganized, successive Physiological States are generated, and (P) influences Physiological Accessibility and downstream behavioral expression (Gomez Uncu, 2026a). Table 5 summarizes their definitions, empirical requirements, and conditions of nonsupport. Organizational processes are not variables in the equations, physiological indicators, latent dimensions, Regulatory Stages, or unifying physiological mechanisms. Nor can they be inferred retrospectively from a behavior change.

### 6.6 Candidate unifying physiological mechanisms

The framework proposes three unifying physiological mechanisms—predictive interoceptive coding, allostatic load redistribution, and Multisystem Threshold Effects (Gomez Uncu, 2025a, 2026a). These mechanisms describe candidate physiological operations contributing, respectively, to anticipatory regulation, cross-system reorganization, and threshold-dependent change. Table 5 distinguishes these mechanisms from the broader Organizational Processes. A mechanism is not an equation variable, a dimension of (P), an indicator, or a Regulatory Stage. Physiological change, an association with (B), or improved in-sample model fit is insufficient to establish a mechanism. Support requires a distinct temporally ordered prediction, relevant comparison models, uncertainty representation, evaluation in separated data, and a prespecified criterion for nonsupport. Evidence supporting one mechanism does not establish either of the others.

## 7 Theoretical predictions and empirical tests

The Nonlinear Physiology–Behavior Equation generates conditional predictions about the evolution of (P) and its prospective relationship with behavioral expression. These predictions follow from three proposed relationships: Physiological Organization generates successive states; interaction between A ↔ P and P ↔ C contributes to the subsequent evolution of (P); and (P) influences Physiological Accessibility, thereby constraining, but not uniquely determining, downstream (B) (Gomez Uncu, 2025a, 2025b, 2026a). Dynamical-systems theory supports the investigation of state dependence, trajectories, persistence, and nonproportional change, but it does not independently validate the framework or its predictions (Kelso, 1995; Strogatz, 2015).

Each empirical test must prespecify the target behavior or functional outcome, the relevant Adaptive Capacity when Physiological Accessibility is examined; the expected relationship; the population and environmental conditions; the timescale; the observation and state-estimation windows; and prediction interval. Prospective tests require a filtered estimate (P̂(t)) based exclusively on information available through time (t), followed by separate measurement of (B(t + Δ)). The target (B) must remain outside (Y(t)), and both state-estimation and parameter uncertainty must be represented.

The minimum comparison set should include a model based on measured (A), relevant prior (C), motivating operations, prior behavior, person-specific variables, and design variables; established stress, arousal, interoceptive, and allostatic constructs or indices; isolated physiological indicators; simple physiological composites; and appropriate linear, lower-dimensional, state-independent, no-history, and nonswitching alternatives. Model development and evaluation must be separated, and all candidate and comparison models must receive comparable opportunities for estimation and tuning. Prospective predictive improvement supports the model’s incremental value but does not, by itself, establish causation, a mechanism through Physiological Accessibility, or any proposed unifying physiological mechanism.

### 7.1 State-dependent behavioral output

Equation 2 predicts that one or more prespecified characteristics of (B), including its probability, form, latency, magnitude, duration, accuracy, or consistency, may vary with (P). The function (f) denotes theoretical dependence; Equation 4 provides the probabilistic formulation. A single state may permit several behavioral responses, and the same response may occur under different states. The empirical test is therefore whether the physiologically estimated state (P̂) provides reproducible prospective and incremental information beyond the minimum comparison set under controlled, standardized, or sufficiently measured environmental conditions.

When the findings are interpreted in terms of Physiological Accessibility, the relevant Adaptive Capacity must be demonstrated independently of the target observations. Variation in behavioral expression across estimated physiological states may be consistent with State-Dependent Accessibility, but it neither directly measures accessibility nor establishes that accessibility mediates the relationship between (P) and (B). Failure of (P̂) to improve held-out prediction, or equal or superior performance by simpler comparison models, would count against the tested state-dependent contribution.

### 7.2 Divergent state trajectories and behavioral outcomes under comparable environmental conditions

Comparable measured values of (A) and relevant prior (C) may be followed by different physiological-state trajectories and behavioral outcomes when they interact with different preceding states or regulatory histories. This prediction is probabilistic and does not presume chaos or mathematical sensitivity to initial conditions. *Comparability* must be operationalized through standardization, prespecified tolerance ranges, matching, stratification, or another justified design. Any unmeasured environmental differences weaken the attribution of trajectory divergence to preceding (P).

Equation 11 permits this prediction to be tested through preceding-state, historical, state-interaction, and threshold terms. To support the proposed trajectory dependence, these terms must improve held-out prediction of subsequent physiological observations, subsequent state evolution, or separately measured (B) beyond models containing environmental main effects, prior behavior, and simpler first-order or longer-memory structures. Visual separation may fall within state-estimation uncertainty.

### 7.3 Stability, instability, recovery, and behavioral expression

Prespecified properties of persistence, instability, or recovery may provide prospective information about subsequent state evolution or (B) beyond the current estimate of (P), mean physiological levels, measured environmental conditions, and simpler temporal models. The framework does not assume that greater stability, stronger coupling, faster recovery, or lower behavioral variability is universally more adaptive. The expected direction of each association must instead be specified for the relevant state region, perturbation, timescale, Adaptive Capacity, and behavioral outcome.

Dynamic predictors must be calculated exclusively from information available before the target outcome. They must also remain analytically distinguishable from the physiological evidence used to estimate the current state. Support requires reproducible improvement beyond current-state, static, stationary, and linear-transition alternatives after accounting for state-estimation uncertainty, artifacts, temporal trends, and environmental change. Failure to return to a defined reference region within a limited observation interval establishes only that recovery was not observed during that interval. It does not establish an absence of recovery or permanent instability.

### 7.4 State-dependent responses to environmental perturbations

The response of (P) to a prespecified environmental perturbation may depend on the preceding state and regulatory history. The perturbations are represented through (A) or relevant prior (C), according to their temporal and functional position, whereas the resulting internal physiological change is presented within Physiological Organization and evolving state. Comparable perturbations may produce different trajectories, and different perturbations may produce similar trajectories. Neither pattern implies that every small demand produces disproportionate physiological change or that a limited response necessarily reflects Regulatory Buffering. Resilience is not an additional framework variable and requires separate operational definition.

A direct model of perturbation type and magnitude is an essential comparator. Repeated within-person designs should specify the perturbation type, intensity, duration, timing, context; the preperturbation state; the response and recovery windows; potential carryover; and the relevant outcomes. Support requires prespecified perturbation-by-state or threshold terms to improve held-out prediction beyond additive and direct-environmental models. Behavioral change alone cannot establish change in (P), Regulatory Buffering, or a Multisystem Threshold Effect.

## 8 Operational hypotheses and model comparisons

The predictions presented in Section 7 yield seven operational propositions concerning physiological measurement, state evolution, Physiological Accessibility, and behavioral output. Table 6 should align each proposition with its corresponding hypothesis, primary tests, comparison models, uncertainty requirements, and criteria for support and nonsupport. Across all propositions, (A) and (C) remain external conditions; (Y) provides partial physiological evidence; (P) is latent; (P̂) is an uncertain model-based estimate; and (B) remains a separately measured outcome. Prospective tests require filtered state estimates and a prespecified separation between model development and evaluation.

**Table 6:** Operational propositions, model comparisons, and decision criteria.

Proposition	Operational hypothesis	Primary test and comparison	Support	Nonsupport
1. Incremental State-Dependent Prediction of Behavioral Expression	A filtered estimate P̂ provides prospective and incremental information about B beyond environmental, historical, established-construct, isolated-indicator, and simpler physiological explanations.	Evaluate the state-related terms in Equation 14 using the null hypothesis in Equation 17. Compare the state-informed model with the minimum comparison set, while retaining the same baseline variables and adding only prespecified P-related terms.	The State-related terms produce calibrated and reproducible improvement in held-out or prospective performance after state and parameter uncertainty are propagated.	The state-related contribution is compatible with the null, does not improve held-out performance, is matched or exceeded by simpler models, or disappears after accounting for artifacts, environmental conditions, prior behavior, temporal trends, and model complexity.
2. Prior-state and history-dependent trajectory prediction	Preceding P̂ and prespecified regulatory history provide prospective information about subsequent P̂ or B beyond current environmental conditions, prior behavior, and simpler temporal models.	Compare Equation 11 with environmental history, no history, first-order, and justified longer memory models containing additional lags, cumulative physiological load, recovery history, or an augmented state. Equation 14 for behavioral output.	Preceding-state terms improve prospective performance; when hypothesized, prespecified longer-memory terms provide additional reproducible value.	First-order terms fail to improve prediction; Failure of both first-order and adequately specified longer-memory models counts against the proposed historical contribution, whereas superiority of a longer-memory model challenges only the first-order approximation.
3. Stability, Instability, Recovery, and Subsequent Expression	Prespecified dynamic properties provide prospective information about subsequent state evolution or B beyond current P̂, average levels, environmental conditions, and simpler temporal models.	Prespecify the reference region, perturbation, windows, expected direction, dynamic features, and outcome; compare with current-state, static, stationary, and linear-transition alternatives while accounting for shared measurement sources. Equations 11, 14, with prespecified dynamic-feature extensions.	The dynamic properties improve held-out prediction beyond current-state and simpler comparison models.	The estimated effects remain within uncertainty, provide no predictive improvement, are matched by static or simpler models, or disappear after artifacts, environmental changes, temporal trends, prior behavior, and complexity are addressed.
4. Threshold-Dependent State and Behavioral Transitions	A prespecified threshold or switching model improves prediction beyond linear transition, smooth nonlinear, and nonswitching alternatives.	Prespecify or develop the threshold index, boundary, window, minimum evidence, and validation procedure. A multisystem threshold effect additionally requires converging multisystem evidence or a validated cross-system feature. Equation 11 for threshold terms; Equation 14 for behavioral output.	The threshold model provides reproducible out-of-sample improvement and the predicted temporal pattern; mechanism claims additionally require multisystem evidence.	The boundary is irreproducible; simpler models perform equally well or better; mechanism support comes only from an isolated indicator or B; or the pattern disappears after artifacts, trends, environmental conditions, and alternative specifications are addressed.
5. Perturbation by State Interaction	Preceding P̂ and a prespecified perturbation-by-state interaction improves prediction beyond perturbation magnitude and additive P effects.	Represent the perturbation through A or relevant prior C; compare direct perturbation, additive perturbation-plus-P, and A-by-P or C-by-P interaction models. Test downstream B separately through an output model. Equations 11, 14.	The interaction improves reproducible held-out prediction beyond additive and direct-environmental models.	The interaction provides no improvement; a direct environmental model performs equally well or better; the effect remains within state estimation uncertainty, or it disappears after carryover, temporal trends, artifacts, environmental differences, and model complexity are addressed.
6. Incremental Value of Multisystem State Estimation	A prespecified multisystem state model improves prediction beyond isolated indicators, established constructs or indices, simple composites, and linear or lower-dimensional states.	Compare an identifiable model integrating evidence from more than one physiologically distinct System or modality with the strongest isolated indicator and other minimum comparators; report coverage, overlap, shared sources, uncertainty, and calibration. Equations 12–15.	Stable and identifiable state estimates produce calibrated out-of-sample improvement beyond the prespecified comparison models.	Simpler models consistently match or exceed performance after complexity, uncertainty, shared sources, and overfitting are addressed.
7. Physiological state and accessibility of existing adaptive capacities	P̂ provides prospective information about the expression of an independently established adaptive capacity without requiring loss and reacquisition of that capacity.	Establish acquisition, mastery, retention, generalization, or criterion performance independently; estimate P̂ before the target expression and compare its incremental value beyond environmental, motivational, historical, task, and simpler physiological models. Equations 14–16.	P̂ predicts reproducible differences in expression while independent evidence indicates that the relevant capacity remains present. Such findings constitute evidence consistent with State-Dependent Accessibility.	P̂ adds no information, or differences are explained better by absence, incomplete acquisition, degradation, insufficient generalization, or environmental and historical alternatives. A P̂–B association alone does not directly measure accessibility or establish mediation.

These propositions operationalize predictions derived from the Nonlinear Physiology–Behavior Equation and related framework constructs (Gomez Uncu, 2025a, 2025b, 2026a). External dynamical system literature supports the general methods and plausibility of state-dependent and nonlinear tests (Kelso, 1995; Strogatz, 2015) but does not establish or validate these framework-specific propositions. Support is restricted to the test population, behavior, context, timescale, indicators, and model specification.

For Proposition 1, Equation 14 provides one candidate behavioral output model containing linear and quadratic state terms. The joint test must account for state-estimation and parameter uncertainty. Its null hypothesis is:


$$H_{0}:\beta =0\;\text{and}\;\Omega =0$$ (17)

Here, 0denotes *a (d)-dimensional zero vector for β* and a (d × d) zero matrix for Ω. Because only the symmetric component of (Ω) is identifiable in the quadratic term, Ω must either be constrained to be symmetric or expressed through unique quadratic and cross-product terms. The state-informed model must retain the variables included in its baseline comparator and add only prespecified (P) -related terms. Rejection of (H₀) is insufficient unless the state-informed model also demonstrates calibrated and reproducible improvement in held-out or prospective data.

Support for any individual proposition is limited to the tested population, behavior, context, timescale, indicators, and operational model test. Repeated failure across adequately measured and appropriately validated operationalizations would challenge the framework’s proposed incremental value. Unsupported findings must not be retrospectively reclassified as evidence of another Physiological State, Regulatory Stage, Organizational Process, or unifying physiological mechanism.

## 9 Implications for measurement and empirical research

The framework provides a structured basis for estimating (P) as a latent, multidimensional, and time-dependent physiological state and evaluating its prospective relationship with (B). The estimate P̂ is derived from physiological observations and features in (Y) and is conditional on a selected indicator, model, population, context, and timescale. It is therefore neither a direct measurement of (P), a validated instrument, nor a universal physiological score. Although (A) and relevant prior (C) may contribute to state evolution, they remain external to (P) and outside (Y); (B) remains a separate downstream outcome. Advanced sensing and computational methods may facilitate estimation, but their use does not independently validate the construct or its proposed interpretation.

### 9.1 Measurement feasibility and Indicator selection

The framework neither prescribes a universal indicator set nor requires every study to measure all Thirteen Physiological Systems and Seven Layers of Physiological State. Indicators should be selected based on theoretical relevance, reliability, sensitivity to within-person state change, temporal compatibility, artifact resistance, nonredundancy, feasibility, and ethical acceptability (Cacioppo et al., 2017). Studies must report their intended System and Layer coverage, including areas of partial or absent coverage and the resulting interpretive limitations. A multisystem estimate requires physiologically distinct evidence intended to inform at least two Systems. Multiple features derived from a single signal do not establish multisystem coverage, and dependencies arising from shared signals, preprocessing procedures, or measurement errors must be represented in the model.

Sampling frequencies, measurement windows, offsets, response delays, and uncertainty representation must correspond to the physiological process under investigation, as formalized in Equation 8. Continuously recorded cardiovascular or electrodermal signals and intermittently sampled endocrine, immune, or metabolic measures are not temporally equivalent. Heart rate variability may provide evidence about cardiac autonomic modulation under appropriate acquisition and analytic conditions, but it is not equivalent to Regulatory Capacity, the Autonomic Layer, or (P) (Task Force of the European Society of Cardiology and the North American Society of Pacing and Electrophysiology, 1996; Laborde et al., 2017; Thayer et al., 2012). Similarly, interoceptive research supports the relevance of internal bodily signaling but does not make self-report measures or task performance physiological indicators of (P) (Craig, 2002; Khalsa et al., 2018). Pilot testing and prespecification should address signal quality, reliability, missing data, artifacts, participant burden, preprocessing, person-specific baselines, indicator dependence, and exclusion criteria. Indicators must not be retained or discarded solely because of their association with (B) in the validation data, because such outcome-guided selection would compromise the independence of the confirmatory test.

### 9.2 Empirical study design and temporal specification

Claims about state evolution require repeated within-person observations. Cross-sectional data may support preliminary indicator selection or observation-model development, but they cannot establish prior-state dependence, recovery, thresholds, or transitions because these properties depend on temporal ordering and within-person change (Kelso, 1995; Strogatz, 2015). Suitable designs include intensive longitudinal studies, repeated-measures studies, controlled-perturbation experiments, N-of-1 studies, and other dynamic latent-variable designs (Asparouhov et al., 2018; Särkkä and Svensson, 2023).

Each study must prespecify the target (B); the relevant Adaptive Capacity when Physiological Accessibility is examined; the measured (A) and relevant prior (C); the physiological observation vector (Y); the sampling, feature-extraction, and state-estimation windows; the prediction interval (Δ), the model family and comparison models; and the criteria for support and nonsupport. In prospective evaluation, the filtered estimate (P̂_r(t_k | t_k)) must use only information available through (t_k), and B_r(t_k + Δ) must be measured during a subsequent interval. Smoothed estimates that incorporate observations obtained after (t_k) are appropriate for retrospective reconstruction but not prospective prediction. The target (B) must remain outside (Y), and the relevant Adaptive Capacity must be established independently of the target observation.

Model development and evaluation must remain separate. Randomly assigning adjacent or overlapping windows to development and evaluation sets may allow shared observations or temporally dependent information to cross the partition, producing temporal leakage. Forward-chaining procedures with nonoverlapping temporal blocks evaluate prediction of later observations within individuals, whereas participant-level holdout evaluates generalization to individuals who did not contribute to model development. Sample-size justification must account for both the number of participants and the number and temporal density of observations per participant. It must also consider autocorrelation, measurement error, missing data, between-person heterogeneity, and the expected frequency of rare transitions. When conventional power analysis cannot adequately represent the proposed latent-state model, simulation-based design analysis targeting a prespecified state recovery accuracy and parameter precision is preferable (Muthén and Muthén, 2002). If the latent dimensions, observation-and-process-error components, or transition parameters cannot be identified with acceptable precision, the model must be simplified (Auger-Méthé et al., 2021; Särkkä and Svensson, 2023). Candidate thresholds and regimes require separate development and evaluation. Controlled-perturbation studies manipulate (A) or relevant prior (C), according to the variable’s temporal and functional role; they do not directly manipulate latent (P). Any intended physiological effect of the perturbation must be demonstrated independently rather than assumed from the intervention assignment.

### 9.3 Computational estimation and reproducibility

Equations 11–16 define three analytically distinct components: the observation model links physiological evidence (Y) to latent (P); the transition model represents the evolution of (P) from its preceding state and permitted external inputs; and the behavioral-output model evaluates separately measured (B). Although (A) and relevant prior (C) may enter the transition model, they remain outside (Y). Likewise, the instance of (B) used to evaluate P̂ cannot contribute to the construction, updating, or selection of that same state estimate.

Candidate implementations include linear and nonlinear state-space models, sequential Monte Carlo or particle-filtering models, hierarchical models, regime-switching methods, and appropriately constrained machine-learning approaches. Standard Kalman filtering assumes linear-Gaussian transition and observation structures and should be used only when assumptions are justified or when it serves as a prespecified comparator model (Kalman, 1960; Särkkä and Svensson, 2023). Greater algorithmic complexity does not establish the existence, dimensionality, or interpretation of (P), nor does it demonstrate distinct states, thresholds, or physiological mechanisms. A model that maps (Y) directly to (B) may predict behavior without estimating (P).

Implementations must report the data sources; sampling and temporal-alignment procedures, preprocessing and missing-data methods; complete model equations, temporal positions of (A), (C), (P̂), and (B); latent dimensionality and identification constraints; process-and observation-error structures; state and parameter uncertainty; estimation procedures and software settings; convergence and diagnostic results; sensitivity analyses; and the separation between development and validation. Evaluation requires held-out physiological observations and, when applicable, separately measured behavioral outcomes; superior in-sample fit is insufficient. Reproducibility should be supported by code or sufficiently detailed pseudocode, software and package versions, dependencies, parameter settings, random seeds, and documented deviations from the prespecified analysis (Sandve et al., 2013).

### 9.4 Validation of the estimated physiological state

Validation must distinguish six targets: indicator adequacy, observation-model validity, temporal validity, construct validity, prospective and incremental value, and generalizability. Table 7 summarizes these validation components and their principal criteria. Validation applies to a specified operationalization rather than to (P) universally, and all validation analyses must propagate state and parameter uncertainty.

**Table 7:** Validation components and Decision Criteria for the Estimated Physiological State.

Validation component	Required evidence	Findings limiting support
Indicator adequacy and physiological coverage	Adequate signal quality, reliability, sensitivity, temporal resolution, and artifact control; explicit System and Layer coverage; dependence among indicators documented.	Poor or unstable measurement; unexplained missingness or artifacts; inadequate temporal resolution; or multisystem claims based on redundant features derived from a single signal.
Observation model and temporal validity	Identifiable relationships between Y and latent P; explicit scale, sign, orientation, and other identification constraints; separation of observation error from process variation; and sufficiently comparable measurement relationships across the relevant times, individuals, devices, and contexts.	Unidentified dimensions; unstable loadings or observation parameters; unaddressed residual dependence or correlated error; measurement relationships that change without being modeled; or apparent dynamics attributable to measurement error, device effects, or parameter drift.
Convergent, discriminant, and perturbational evidence	Convergence from independent physiological evidence when predicted; distinction from isolated indicators, stress, arousal, allostatic load, interoception, emotion, and other narrower constructs; reproducible response to prespecified environmental perturbation.	Agreement limited to redundant transformations of the same signal; construct equivalence inferred from correlation alone; or perturbation-related findings explained adequately by artifacts, direct environmental effects, temporal trends, or simpler response models.
Prospective and incremental validity	Filtered P̂ improves held-out prediction beyond A, prior C, motivating operations, prior B, isolated indicators, established constructs, composites, and linear or lower-dimensional alternatives; outcome-appropriate calibration and performance.	No reproducible incremental value; equal or superior performance by simpler models; inadequate calibration; temporal or target leakage; or apparent improvement attributable to unpropagated state or parameter uncertainty.
Generalization and robustness	Temporally structured internal validation; participant-level and external validation for the corresponding claims; robustness across reasonable preprocessing, windows, missing-data assumptions, dimensionalities, and transition model specifications.	Failure to generalize across later observations, participants, populations, devices, settings, or other conditions included in the claim; material sensitivity to reasonable analytical choices; or poor calibration despite adequate discrimination.

These components adapt established principles of psychophysiological measurement, measurement comparability, predictive validation, and state-space estimation (Putnick and Bornstein, 2016; Cacioppo et al., 2017; Steyerberg, 2019; Särkkä and Svensson, 2023). They define procedures for validating a specified operationalization of P(t) but do not independently establish latent P or its framework-specific interpretation.

Including (A) or prior (C) in (Y) cannot establish physiological measurement validity because these variables are external to (P). Similarly, the target (B) cannot be used to construct or update the (P̂) evaluated against that outcome. When Physiological Accessibility is examined, behavioral nonexpression alone cannot establish restricted accessibility. No individual indicator, latent dimension, fit statistic, or behavioral prediction can validate (P) by itself. Unidentifiable dimensions, unstable measurement relationships, dynamics that cannot be distinguished from observation error or simpler alternatives, and failure to demonstrate reproducible prospective and incremental value would count against the tested operationalization.

### 9.5 Implications for prediction and state-informed intervention

Prediction, intervention selection, and causal explanation constitute three different levels of evidence. A filtered estimate of (P) may improve prediction of (B) without demonstrating that decisions based on the estimate improve outcomes. A state-informed decision rule with prespecified actions and benefit criteria must therefore be compared with fixed, environmental-only, prior-behavior, and simpler physiological rules and evaluated in terms of functional benefit, participant burden, and potential harm. (P) is not directly observed and should not be treated as an independently manipulable intervention target. Interventions act on environmental conditions, demands, supports, Adaptive Capacities, or independently established physiological conditions; (P) may then be re-estimated to determine whether the intervention was followed by a state-related change.

Establishing a causal pathway through (P) requires temporal ordering, an appropriate experimental or causal-inference design, propagation of state and parameter uncertainty, and explicit evaluation of confounding and alternative pathways (VanderWeele, 2015; Hernán and Robins, 2020). State-informed approaches should complement, not replace, behavioral intervention, communication support, skill development, accommodation, rehabilitation, or medically indicated care. They must not treat (P̂) as a diagnosis, severity score, measure of personal worth, or justification for restricting participation or support. Physiological normalization is not the objective.

The required evidence depends on the proposed application. Prediction requires prospective incremental performance. Intervention selection requires a state-informed rule to improve outcomes relative to a nonstate-informed rule without imposing unacceptable burden or harm. A causal pathway requires reproducible state-related change that precedes the outcome, together with a design capable of addressing confounding and plausible alternative pathways. Failure at one level counts against that application but does not automatically determine the validity of the others. Allostatic research supports the plausibility of state-dependent physiological responses, but it does not establish the efficacy or mechanism of a framework-specific intervention (McEwen, 1998; Sterling, 2012).

## 10 Discussion

The framework proposes a nonlinear, state-dependent system in which external (A) and relevant prior (C) contribute to the evolution of (P), while current (P) may modulate their functional effects across time. The bidirectional relationships are a theoretical claim about organism–environment coupling; the present transition equations model the evolution of (P) rather than the separate evolution of (A) or (C). (B) remains the downstream behavioral outcome. (P) is the latent physiological state generated by ongoing Physiological Organization. Through Physiological Accessibility, it is proposed to constrain, without uniquely determining, the recruitment and behavioral expression of existing Adaptive Capacities. Across longer timescales, (P) may also influence the physiological conditions under which new Adaptive Capacities are acquired or strengthened. This longitudinal development pathway is distinct from the momentary accessibility of existing capacities and requires separate empirical evaluation (Gomez Uncu, 2025a, 2025b, 2026a).

The framework contribution is not a new sensor, biomarker, or estimation algorithm. Rather, it provides a measurement-development architecture that specifies physiological evidence in (Y); observation and transition models; filtered estimation of (P̂), representation of uncertainty; prospective behavioral output testing; comparison models; validation criteria; reproducibility requirement; and explicit conditions of nonsupport. The framework remains theoretical and methodologically developmental. It is not a validated instrument, diagnostic system, universal physiological score, or established causal mechanism.

### 10.1 Relationship to existing behavioral and physiological models

The framework complements rather than replaces functional accounts of antecedent conditions, consequences, motivating operations, stimulus control, reinforcement, and learning history (Skinner, 1953; Michael, 1993; Laraway et al., 2003; Cooper et al., 2020). Its additional, testable hypothesis is that an independently estimated physiological state (P̂) provides reproducible prospective and incremental information about subsequent (B) beyond measured environmental conditions and behavioral history. Residual behavioral variation after accounting for these variables does not constitute evidence of (P); it may instead reflect unmeasured environmental conditions, prior behavior, measurement error, inadequate sampling, or model misspecification.

Integrative physiology and psychophysiology support the measurement of coordination of physiological processes across systems (Hall, 2016; Cacioppo et al., 2017). Allostatic models describe adaptive, anticipatory, and history-dependent regulation (McEwen, 2000; Sterling, 2012), whereas interoceptive research examines the signaling and representation of internal bodily conditions (Craig, 2002; Khalsa et al., 2018). These bodies of research establish the plausibility of relevant physiological processes but do not validate the framework-specific construct of (P). (P) must not be treated as synonymous with physiology generally, stress, arousal, allostatic load, or interoception. Dynamical-systems theory provides concepts for describing trajectories, persistence, nonproportional change, and state transitions, while state-space methods provide tools for estimating latent states from repeated, incomplete, and noisy observations (Kelso, 1995; Strogatz, 2015; Auger-Méthé et al., 2021; Särkkä and Svensson, 2023). These theories and methods support the proposed analytical strategy, but they do not establish the existence, dimensionality, physiological interpretation, or behavioral relevance of (P). Linear, nonlinear, threshold, and regime-switching specifications must therefore be compared empirically rather than treated as properties of the framework by definition.

### 10.2 Proposed theoretical and methodological contributions

The proposed novelty lies in the integration and analytical ordering of existing theoretical and methodological foundations within the Nonlinear Physiology–Behavior Equation. The framework distinguishes the continuous process of Physiological Organization from the latent state (P), its empirical estimate (P̂), Physiological Accessibility, and existing Adaptive Capacities. It simultaneously preserves the analytical separation of external (A) and relevant prior (C), physiological evidence (Y), and downstream (B). The framework also distinguishes the Thirteen Physiological Systems, Seven Layers of Physiological State, dynamic properties, Regulatory Stages, Organizational Processes, and Unifying Physiological Mechanisms. These categories serve different theoretical and analytical functions; none automatically corresponds to an indicator, an equation variable, or a latent dimension of (P).

This architecture makes the framework empirically accountable. An operationalization of (P̂) must be derived from physiological evidence, accompanied by model-appropriate uncertainty, evaluated prospectively, and compared with environmental, historical, established-construct, isolated-indicator, simple-composite, linear, and lower-dimensional alternatives. Although complete coverage of every System or Layer is not required, studies must report the intended coverage, overlap among indicators, areas of partial or absent coverage, and limitations these impose on interpretation. Clinical observations may have motivated the hypotheses, but they do not validate the proposed constructs or relationships. Repeated failure of theoretically justified and adequately measured operationalizations to produce identifiable, reliable, and incrementally useful estimates of (P̂) would challenge the framework.

### 10.3 Limitations of the current framework

The first limitation is the absence of a directly observable gold standard for (P). Every estimate (P̂) is conditional on the selected indicators, measurement windows, preprocessing procedures, identification constraints, population, behavior, timescale, and context. No universal dimensionality, indicator set, or minimum System or Layer coverage has been established. Moreover, shared signals and preprocessing procedures may induce associations between (P̂) and measures of Physiological load, Regulatory Capacity, variability, recovery, or coupling, even when these measures do not provide independent evidence. The second limitation concerns computational identifiability. Nonlinear state-space models may be weakly identified, unstable, overfitted, or unable to distinguish latent process variation from observation error. Artifacts, movement, medication, sleep, illness, biological rhythms, missing data, irregular sampling, and unmeasured environmental conditions may produce apparent persistence, thresholds, or transitions. Model convergence or the detection of a cluster, change point, or regime, therefore, does not establish a Physiological State or a Multisystem Threshold Effect (Auger-Méthé et al., 2021; Muthén and Muthén, 2002; Särkkä and Svensson, 2023).

The third limitation concerns causal interpretation. The A↔ P ↔ C representation is a conceptual dependency architecture rather than a complete causal model. Prospective prediction by (P̂) would not establish that (P) caused (B), mediated an intervention effect, or would alter behavior if manipulated (VanderWeele, 2015; Hernán and Robins, 2020). Social, cultural, developmental, symbolic, contextual, historical, and learning-related influences remain analytically distinct from (P). Inadequate measurement or representation of these influences could result in their effects being incorrectly attributed to physiology. Physiological Accessibility is also unobserved; its interpretation requires independent evidence that the relevant Adaptive Capacity exists and cannot be inferred solely from behavioral nonexpression.

The fourth limitation concerns generalizability and practical feasibility. Measurement relationships and state dynamics cannot be assumed to generalize across individuals, populations, behaviors, devices, settings, or timescales. Multimodal monitoring may be costly, burdensome, invasive, or systematically incomplete. It also raises concerns about informed consent, privacy, surveillance, data security, incidental findings, unequal measurement performance, and algorithmic bias. Estimates (P̂) must not be used as diagnosis, severity score, measure of personal worth, or justification for restricting communication, education, participation, services, or autonomy. Independent replication is particularly important because the framework substantially develops constructs introduced in the author’s previous theoretical work.

### 10.4 Priorities for empirical development

Empirical development should proceed through progressively stronger phases, as summarized in Table 8. Initial studies should focus on a limited target behavior, population, timescale, and theoretically justified set of physiological indicators. Failure to demonstrate incremental prospective value should preclude claims of clinical or decision-making utility. Failure of nonlinear models to outperform appropriate linear alternatives should constrain the corresponding nonlinear claims without automatically rejecting simpler state representations. Transparent reporting, preserved computational workflows, fair model comparisons, sensitivity analyses, and independent replication should allow the framework to be supported, constrained, revised, or rejected (Sandve et al., 2013).

**Table 8:** Phased priorities and advancement criteria for empirical development.

Phase	Primary requirements	Advancement or stopping criteria
1. Measurement feasibility and state identification	Prespecify the target B, relevant adaptive capacity when applicable, population, A, prior C, timescale, and physiological coverage. Evaluate signal quality, reliability, within-person sensitivity, temporal resolution, artifacts, missingness, burden, nonredundancy, observation models, dimensionality, error structure, identification constraints, and uncertainty.	Advance only if P̂ is identifiable, physiologically interpretable, sufficiently stable, and not reducible to an artifact, isolated indicator, or simple composite. Otherwise, revise or reject that operationalization.
2. Temporal and state-transition validity	Use repeated observations to evaluate persistence, change, recovery, and transitions beyond observation error, physiological rhythms, artifacts, overlapping windows, sampling irregularities, and environmental differences. Compare prior-state and history-dependent models with environmental-only, no-history, and linear-transition alternatives; evaluate parameter recovery when required by model complexity.	Advance only when temporal structure reproduces in held-out data and improves on simpler models. Failure to distinguish process variation from observation error or to recover essential transition parameters requires model simplification or rejection of the tested transition specification.
3. Prospective behavioral validity and physiological accessibility	Estimate filtered P̂ using only information available through the prediction time and compare the prediction of subsequent B with the minimum comparison set in Section 7. Establish the relevant adaptive capacity independently before testing state-dependent accessibility. Acquisition or strengthening of a new capacity requires a separate longitudinal analysis.	Advance when P̂ produces meaningful, calibrated, out-of-sample incremental value. Equal or superior performance by simpler models limits the claimed contribution of P. Failure to establish the relevant capacity precludes an interpretation based on Physiological Accessibility.
4. Nonlinear propositions, organizational processes, and unifying physiological mechanisms	Test prespecified prior-state interactions, perturbation-by-state relationships, recovery dynamics, coupling patterns, and threshold regions against additive, linear, smooth nonlinear, no-history, and nonswitching alternatives. Operationalize each organizational process and unifying physiological mechanism separately.	Advance each claim only when its prespecified pattern reproduces and outperforms the relevant alternatives. Evidence for one proposition, organizational Process, or Unifying Physiological Mechanism does not establish the others. Failure of a nonlinear term to outperform its comparator constrains that term rather than every state representation.
5. External validation, independent replication, and clinical utility	Apply previously developed indicators, preprocessing, models, and prediction rules across separated participants, populations, behaviors, settings, devices, and timescales. Compare state-informed decisions with appropriate non-state-informed rules and evaluate safety, burden, feasibility, and ethics. Causal or mediation claims require appropriate designs and uncertainty propagation.	Clinical claims require external validity and improvement in meaningful outcomes without unacceptable harm or burden. Failure of the decision rule or causal test limits that application and must not be reclassified as support for another mechanism.

The phases are conceptually ordered but may be iterative within model development. Advancement to a later phase does not eliminate the need to reassess earlier requirements when indicators, populations, devices, contexts, or model specification change. The methods draw on psychophysiological measurement, parameter-recovery assessment, state-space modeling, predictive validation, causal inference, and reproducible research (Muthén and Muthén, 2002; Sandve et al., 2013; VanderWeele, 2015; Cacioppo et al., 2017; Steyerberg, 2019; Hernán and Robins, 2020; Särkkä and Svensson, 2023). These sources support the methods but do not validate the framework-specific constructs or their proposed relationships.

## 11 Conclusion

This work proposes an operational framework in which (P) is represented as a latent, multidimensional, and time-dependent physiological state generated by ongoing Physiological Organization. Its model-based empirical estimate (P̂) is derived from temporally aligned physiological observations and dynamic physiological features. It is not a direct measurement of the underlying state.

The contribution is neither a new sensor or universal physiological score nor the general claim that physiology influences behavior. Rather, it is an integrated measurement-development framework that distinguishes Physiological Organization, (P), (P̂), Physiological Accessibility, and Adaptive Capacities while preserving the analytical separation of external (A), relevant prior (C), physiological evidence (Y), and downstream (B). The framework specifies construct boundaries, indicator selection, temporal alignment, dynamic feature derivation, latent-state estimation, uncertainty representation, model comparison, prospective validation, and reproducibility.

The framework generates testable propositions concerning state-dependent behavioral expression, prior-state dependence, recovery, perturbation-by-state interactions, candidate threshold regions, multisystem estimation, and the expression of existing Adaptive Capacities across physiological states. Testing these propositions requires a filtered estimate (P̂) constructed independently of the target (B), explicit representation of uncertainty, and comparison with relevant environmental, historical, established-construct, isolated-indicator, simple-composite, linear, and lower-dimensional alternatives.

The framework remains theoretical and methodologically developmental. It is not a validated instrument, diagnostic system, established causal explanation, or intervention protocol. Support would require (P̂) to provide reproducible prospective and incremental information about (B). When the relevant Adaptive Capacity has been established independently, repeated state-related differences in its expression may provide evidence consistent with, but not direct measurement of, variation in Physiological Accessibility. If (P̂) cannot be obtained reliably, distinguished from measurement error and simpler physiological representations, or shown to provide information beyond appropriate comparison models, the tested operationalization would not be supported. Repeated failure across theoretically justified, adequately measured, and independently evaluated operationalizations would challenge the broader framework. Its scientific value must therefore be determined by the cumulative empirical evidence.
